# Temporal and genotype-dependent transcriptomic responses to cold treatment and recovery in winter rapeseed (*Brassica rapa* L.)

**DOI:** 10.3389/fpls.2026.1947775

**Published:** 2026-09-17

**Authors:** Yifan Wang, Yuanyuan Zhang, Shiyi Li, Hao Sun, Zhengnan Xu, Lijun Liu, Tun Liu, Junyan Wu, Gang Yang, Wancang Sun, Li Ma

**Affiliations:** State Key Laboratory of Aridland Crop Science, College of Agronomy, Gansu Agricultural University, Lanzhou, China

**Keywords:** candidate genes, cold treatment and recovery, comparative transcriptomics, genotype-by-treatment interaction, reference genome selection, winter rapeseed

## Abstract

Winter rapeseed (*Brassica rapa* L.) is exposed to recurrent low-temperature episodes, yet cultivar differences and reference-genome choice can complicate the interpretation of transcriptomic variation. Here, we reanalyzed 24 growth-point RNA-seq samples from the cold-tolerant cultivar ‘Longyou-7’ and the cold-sensitive cultivar ‘Lenox’ under control conditions (CK), after 3 and 24 h at 4 °C, and after 24 h of recovery at 22 °C. Transcriptome analysis was performed using the chromosome-level Longyou-7 reference genome and compared with results obtained using Chiifu v1.5. Functional analyses revealed stage-dependent transcriptional changes involving photosynthesis, lipid metabolism, hormone signaling, circadian regulation, sulfur and amino acid metabolism, and other stress-related processes. Glutathione metabolism was represented among the top-ranked pathways in several within-genotype contrasts, and genotype-specific co-expression networks linked glutathione-related transcription with antioxidant enzyme activities, soluble sugar, and ROS-associated traits. Biochemical measurements further showed stage-dependent changes in GSH and GSSG contents and GST activity, with ‘Longyou-7’ maintaining a higher GSH/GSSG ratio and higher GST activity than ‘Lenox’ during cold treatment. qRT-PCR analysis of 22 candidate genes showed overall concordance with RNA-seq (*R*² = 0.736), and 17 candidates contained conserved GST_N and GST_C domains, including nine Tau and eight Phi GSTs. Relative to Chiifu v1.5, the Longyou-7 reference increased uniquely mapped read rates by 1.28–1.69 percentage points in Longyou-7 samples, whereas differences in ‘Lenox’ ranged from −0.26 to 0.30 percentage points, while shared DEG responses remained highly concordant between the two reference backgrounds. Together, these results reveal stage-dependent transcriptional and biochemical adjustment during cold treatment and recovery and identify GSH/GST-related genes as candidates for further functional analysis in winter rapeseed.

## 1 Introduction

Winter rapeseed (*Brassica rapa* L.) is an important overwintering oilseed crop in the cold, high-altitude regions of northwestern China. Its shoot apices can survive severe winter conditions and resume growth as temperatures rise in the following year (Sun et al., 2011; Wu et al., 2022b). The cold-tolerant cultivar ‘Longyou-7’ (Longyou-7) and the cold-sensitive cultivar ‘Lenox’ differ markedly in field overwintering ability, morphology, and physiological responses to low temperature (Ma et al., 2019; Wu et al., 2022a). Previous physiological, biochemical, proteomic, and transcriptomic studies have implicated signal transduction, osmotic adjustment, membrane protection, and antioxidant defense in winter-rapeseed cold responses (Ma et al., 2019; Liu et al., 2022; Wu et al., 2022b). However, direct comparisons between cultivars at a treatment stage combine constitutive genotype differences with differences in treatment response. A factorial analysis that explicitly tests genotype × treatment-stage interactions is therefore required to identify transcriptional responses that differ between cultivars.

Low temperature is a major abiotic stress that restricts plant growth and development, geographical distribution, and crop productivity (S. Sanghera et al., 2011; Xu et al., 2026). Freezing and chilling injuries can reduce membrane fluidity, disrupt photosynthetic and respiratory electron transport, and promote the accumulation of reactive oxygen species (ROS). When ROS production exceeds cellular scavenging capacity, ROS can promote membrane lipid peroxidation and damage proteins and nucleic acids (Hasanuzzaman et al., 2020; Huang et al., 2022). Plants therefore maintain cellular homeostasis through interconnected processes that include low-temperature perception and signal transduction, transcriptional regulation, osmotic adjustment, membrane lipid remodeling, and antioxidant defense (Ding et al., 2019; Liu et al., 2022; Wang et al., 2024). Because these processes operate on different timescales, comparisons across early cold exposure, prolonged treatment, and post-treatment recovery can distinguish transient transcriptional responses from changes that persist or emerge later.

RNA sequencing (RNA-seq) results reflect not only biological differences among samples but also the reference sequence and gene annotation used for analysis (Ritonga and Chen, 2020). In particular, sequence similarity between the reference genome and the genotype under investigation can affect read mapping, expression quantification, and candidate gene identification (Zhang et al., 2018). Transcriptomic studies of Brassica species have long relied on the reference genome of the Chinese cabbage accession Chiifu-401-42, whose assembly and annotation have undergone multiple updates (Zhang et al., 2023). Brassica germplasm, however, exhibits extensive morphological and ecotypic diversity, and a single reference sequence cannot represent the full range of structural and gene-content variation within the species (Hannah et al., 2005; Bayer et al., 2020). When the reference material and the experimental genotype differ in sequence content, read mapping, estimated expression levels, and the composition of candidate gene sets may vary with the selected reference and annotation; the magnitude of these effects must therefore be evaluated for the dataset being analyzed (Brown et al., 2017; Coombes et al., 2024).The chromosome-level Longyou-7 reference genome was generated from the cold-tolerant cultivar used in the present study. It therefore provides genotype-matched sequence and annotation coordinates for Longyou-7 samples and a material-specific framework for analyzing candidate genes in winter rapeseed (Wu et al., 2022a). A direct reanalysis of the same RNA-seq dataset against Longyou-7 and Chiifu v1.5 can separate biological comparisons between the two cultivars from changes attributable to the analytical reference background. Such a comparison does not by itself establish reference bias or prove that one reference is optimal for every genotype, but it can identify differences in mapping performance, annotated gene correspondence, and the representation of candidate genes.

Plant cells possess a coordinated antioxidant defense system composed of enzymatic and nonenzymatic components. The GSH metabolism pathway is a central part of this system (Ghanta et al., 2011; Rai et al., 2023). GSH is a tripeptide composed of glutamate, cysteine, and glycine and is the most abundant non-protein thiol in plant cells (Zeng et al., 2012). It contributes to cellular redox buffering and can be conjugated with electrophilic compounds or products generated under oxidative stress through GST-catalyzed reactions. Reduced GSH also participates in the ascorbate–glutathione cycle, glutathione peroxidase-catalyzed reactions, and GST-mediated detoxification. Together, these processes contribute to ROS scavenging and cellular redox homeostasis, making GSH an important component of the plant stress-detoxification network (Hasanuzzaman et al., 2019, 2020; Zhang et al., 2025).

Accordingly, this study reanalyzed 24 growth point tissue RNA-seq samples from ‘Longyou-7’ and ‘Lenox’ under the control condition (CK), after cold treatment at 4 °C for 3 h (3 h) or 24 h (24 h), and after 24 h of recovery following the 24-h cold treatment (Re24h). Expression quantification and functional analyses used the chromosome-level Longyou-7 reference genome, and mapping performance, annotated-gene correspondence, and candidate-gene representation were compared with results based on Chiifu v1.5. Differential expression was evaluated with a factorial model containing genotype, treatment stage, and their interaction, together with within-genotype cold and recovery contrasts. WGCNA was performed separately for the two genotypes, and module–trait relationships were evaluated within each genotype across treatment and recovery stages. GSH- and GST-related genes were evaluated as transcript-level candidates and assessed by qRT-PCR.

## 2 Materials and methods

### 2.1 Plant materials, cold treatments, and growth point tissue collection

The cold-tolerant winter rapeseed cultivar ‘Longyou-7’ and the cold-sensitive cultivar ‘Lenox’ were used in this study. Plants were cultivated and subjected to cold treatment as described by Ma et al. (2019). Seeds were germinated on moist filter paper, and radicle emergence was observed on day 3. Seedlings were transplanted into nutrient soil on day 10 and maintained at 22 °C. At 35 days after sowing, plants maintained at 22 °C were used as the control group (CK). For cold treatment, plants were transferred to 4 °C for 3 or 24 h. For the recovery treatment (Re24h), plants were exposed to 4 °C for 24 h and subsequently returned to 22 °C for an additional 24 h. Each cultivar-by-stage combination included three independent biological replicates, with one individually potted plant per replicate, yielding 24 samples in total.

### 2.2 Physiological and biochemical measurements

frozen growth-point tissues using an H_2_O_2_ assay kit (cat. no. BC3590) and an O_2_^-^ assay kit (cat. no. BC1290), respectively (Beijing Solarbio Science & Technology Co., Ltd., Beijing, China), according to the manufacturer’s instructions. Reduced glutathione (GSH) content, oxidized glutathione (GSSG) content, and glutathione S-transferase (GST) activity were determined in growth-point tissues using commercial assay kits (cat. nos. AKPR008M, AKPR009M, and AKPR013M, respectively; Beijing Boxbio Science & Technology Co., Ltd., Beijing, China). The GSH/GSSG ratio was calculated separately for each biological replicate from the corresponding GSH and GSSG contents. Three independent biological replicates were analyzed for each cultivar-by-stage combination. Peroxidase (POD) and catalase (CAT) activities and soluble sugar (SS) and malondialdehyde (MDA) contents were obtained from Ma et al. (2019). For WGCNA module–trait association analysis, POD, CAT, SS, MDA, H_2_O_2_, and O_2_^-^ measurements were matched to the corresponding RNA-seq samples by cultivar, treatment stage, and biological replicate.

### 2.3 RNA extraction, library construction, and reanalysis of the original reads

Total RNA was originally extracted from growth point tissues using TRIzol reagent (Invitrogen, Carlsbad, CA, USA). RNA purity and concentration were assessed using a NanoDrop 2000 spectrophotometer (Thermo Scientific, Wilmington, DE, USA), and RNA integrity was evaluated using an Agilent 2100 Bioanalyzer (Agilent Technologies, Santa Clara, CA, USA). Poly(A)^+^ RNA libraries were constructed and quality checked as described by Ma et al. (2019) and sequenced by Shanghai OE Biotech Co., Ltd. (Shanghai, China) on an Illumina HiSeq X Ten platform to generate 150-bp paired-end reads. The present study generated neither new libraries nor new sequencing data; the original 2018 FASTQ files were reprocessed against the Longyou-7 reference genome.

### 2.4 Transcriptome data processing, differential expression, and functional analysis

Adapter sequences and low-quality reads were removed using fastp (Chen et al., 2018). Clean reads were aligned to the chromosome-level Longyou-7 reference genome (Wu et al., 2022a) using HISAT2 (Kim et al., 2015), and raw gene counts were obtained with HTSeq-count (Anders et al., 2015). FPKM values (Roberts et al., 2011) were used for descriptive expression displays and the original WGCNA input; differential expression was evaluated from raw counts. Genes with a count of at least 10 in at least three samples were retained. A negative-binomial generalized linear model was fitted in PyDESeq2 version 0.5.4 (Love et al., 2014; Muzellec et al., 2023) with genotype, treatment stage, and genotype × treatment-stage interaction as design terms; ‘Lenox’ and CK were the reference levels. Within each genotype, 3 h, 24 h, and Re24h were contrasted with CK, and Re24h was contrasted with 24 h to characterize recovery. Formal interaction contrasts tested whether the response from CK to each stage differed between genotypes; an additional difference-in-differences contrast tested whether the Re24h-versus-24 h recovery change differed between genotypes. Stage-matched Longyou-7-versus-Lenox contrasts were retained only as descriptive, baseline-sensitive comparisons. DEGs were defined by a Benjamini–Hochberg-adjusted p-value < 0.05 and |log_2_ fold change| ≥ 1. GO (The Gene Ontology Consortium, 2019) and KEGG (Kanehisa et al., 2007) enrichment used the tested genes as background and Benjamini–Hochberg correction. For reference-genome comparison, Longyou-7 and Chiifu v1.5 analyses must use identical preprocessing, count filters, model contrasts, DEG thresholds, enrichment backgrounds, and multiple-testing procedures; sequence-similarity matches alone were not treated as proof of orthology or function. To assess the influence of reference-genome choice on DEG detection, differential-expression results obtained using the Longyou-7 and Chiifu v1.5 reference genomes were re-filtered using the same criteria of adjusted P < 0.05 and |log_2_ fold change| ≥ 1. Longyou-7 gene models were linked to retained Chiifu v1.5 targets using one-direction BLASTn best-hit mapping. DEG calls were summarized as shared, Longyou-7-reference-specific, or Chiifu-reference-specific best-hit groups, and overlap was quantified using the Jaccard index. For shared best-hit DEG pairs, expression-direction concordance and Pearson correlation of log_2_ fold-change values were calculated. Longyou-7-reference DEGs without a retained Chiifu v1.5 BLAST hit were additionally examined for functional annotation and KEGG enrichment. A no-hit DEG therefore indicates the absence of a retained BLAST match under the applied comparison settings and was not interpreted as evidence of genomic absence from Chiifu.

### 2.5 Expression-pattern clustering and weighted gene co-expression network analysis

For WGCNA, separate co-expression networks were constructed for ‘Longyou-7’ and ‘Lenox’ using the 12 RNA-seq samples from each genotype. FPKM values were transformed as log_2_(FPKM + 1), and genes with a standard deviation ≤ 0.5 within each genotype were removed. Candidate soft-thresholding powers from 1 to 30 were evaluated separately for the two networks, and the selected powers were determined according to scale-free topology criteria. Adjacency and topological overlap matrices were generated independently for each genotype, followed by dynamic tree cutting and module merging. Within each genotype, Pearson correlations were calculated between module eigengenes and POD, CAT, SS, MDA, H_2_O_2_, and O_2_^-^ values across the corresponding 12 samples. Module eigengene profiles were summarized across CK, 3 h, 24 h, and Re24h within each genotype. Module colors were assigned independently in the two networks and therefore do not indicate corresponding modules between cultivars. For the Longyou-7 coral1 and darkslateblue modules, the top 10 hub genes were ranked using the maximal clique centrality (MCC) algorithm in cytoHubba, and simplified co-expression subnetworks retaining the connections among these genes were visualized in Cytoscape.

### 2.6 Candidate gene selection and qRT-PCR assessment

A total of 61 candidate genes were identified by intersecting genes assigned to the GO term glutathione metabolic process with those assigned to the KEGG pathway glutathione metabolism. The qRT-PCR validation set comprised 22 genes, including 15 high-abundance candidates selected according to the highest mean FPKM values across the 24 RNA-seq samples, six low-abundance candidates, and one Longyou-7-reference-specific annotated candidate. For low-abundance selection, the 61-gene set was intersected with the 39 glutathione metabolism-related genes represented in **Figures 1** and **2**; genes with an FPKM of zero in any sample were excluded, and the six genes with the lowest mean FPKM values were retained. Gene-specific primers were designed from CDS sequences using NCBI Primer-BLAST. Total RNA was extracted using the RNA Easy Fast Plant Tissue RNA Rapid Extraction Kit (DP452, TIANGEN, Beijing, China), and 1 μg RNA was reverse-transcribed using FastSensi One-Step Genomic DNA Removal and First-Strand cDNA Synthesis SuperMix (KR138, TIANGEN). qRT-PCR was performed using FastReal Fast Quantitative PCR PreMix (FP217, TIANGEN), with *ACTIN* as the reference gene. Relative transcript abundance was calculated using the 2^^−ΔΔCt^ method (Moebes et al., 2022; Pathak et al., 2024). Three independent biological replicates, each with three technical replicates, were analyzed for each cultivar-by-stage combination. Among the 22 validated candidates, 17 proteins contained both GST_N and GST_C domains and were classified into Tau and Phi subclasses using the NCBI Conserved Domain Database. Conserved motifs were identified using MEME, and phylogenetic relationships, motifs, and domain architectures were visualized using TBtools-II. The same analyses were performed for 26 GST-related proteins retained from the Chiifu v1.5 reference background after conserved-domain screening. For broader comparison, a phylogenetic tree was constructed using 126 GST-related protein sequences from *Arabidopsis thaliana*, Chiifu, Longyou-7, and *Brassica napus*.

**Figure 1:**
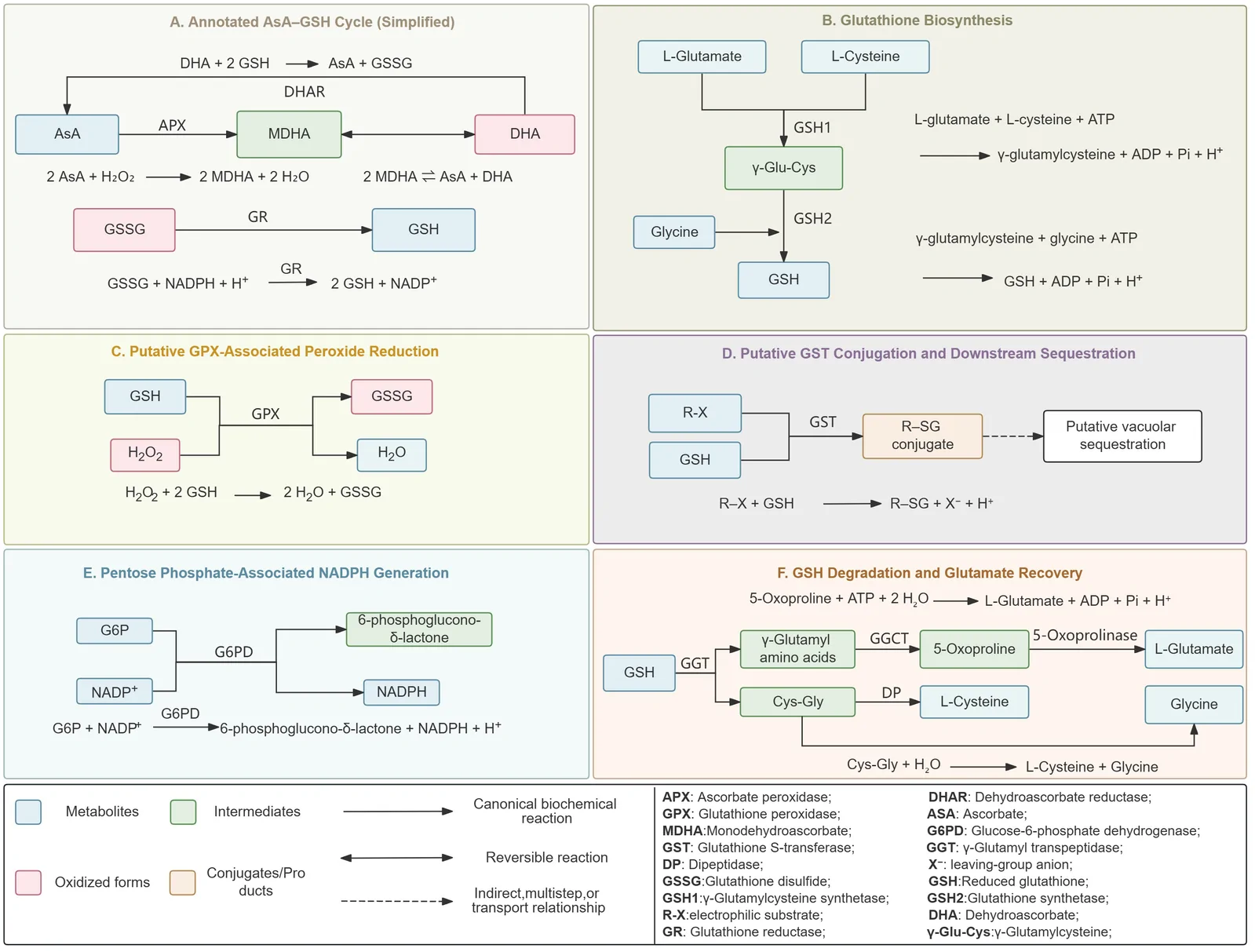
Schematic representation of annotated glutathione-related metabolic processes. The diagram summarizes **(A)** the simplified ascorbate–glutathione (AsA–GSH) cycle, **(B)** annotated reactions involved in glutathione biosynthesis, **(C)** a putative glutathione peroxidase (GPX)-associated peroxide-reduction reaction, **(D)** putative glutathione S-transferase (GST)-mediated conjugation and downstream sequestration, **(E)** pentose phosphate pathway-associated NADPH generation, and **(F)** GSH degradation and glutamate recovery. Metabolites, intermediates, oxidized forms, and conjugates or products are distinguished by color, whereas enzymes are indicated adjacent to the reactions they catalyze. Solid single-headed arrows indicate canonical biochemical reactions, double-headed arrows indicate reversible reactions, and dashed arrows indicate indirect, multistep, or transport relationships. APX, ascorbate peroxidase; AsA, ascorbate; DHA, dehydroascorbate; DHAR, dehydroascorbate reductase; DP, dipeptidase; G6PD, glucose-6-phosphate dehydrogenase; GGCT, γ-glutamylcyclotransferase; GGT, γ-glutamyl transpeptidase; GPX, glutathione peroxidase; GR, glutathione reductase; GSH, reduced glutathione; GSSG, glutathione disulfide; GST, glutathione S-transferase; GSH1, γ-glutamylcysteine synthetase; GSH2, glutathione synthetase; MDHA, monodehydroascorbate; γ-Glu-Cys, γ-glutamylcysteine; and X^-^, a leaving-group anion.

**Figure 2:**
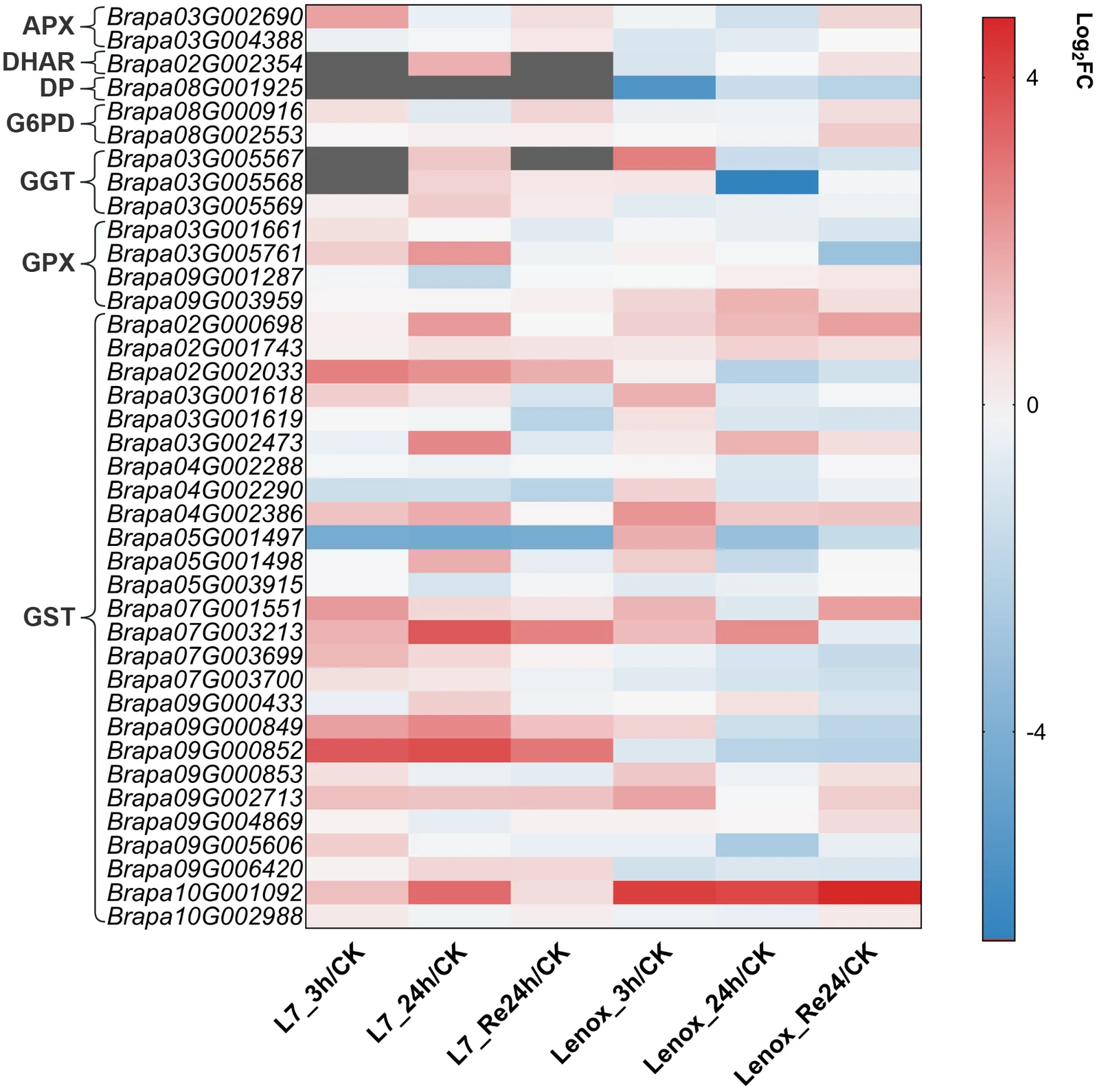
Transcript-level responses of genes annotated to glutathione-related processes during cold treatment and recovery in ‘Longyou-7’ and ‘Lenox’. The heatmap shows within-genotype log_2_ fold changes (log_2_FC) for each treatment relative to the corresponding genotype-matched untreated control (CK). Columns represent ‘Longyou-7’ after 3 h and 24 h at 4 °C and after 24 h of recovery at 22 °C following 24 h of cold treatment (Longyou-7_3h/CK, Longyou-7_24h/CK, and Longyou-7_Re24h/CK, respectively), followed by the corresponding contrasts for ‘Lenox’ (Lenox_3h/CK, Lenox_24h/CK, and Lenox_Re24h/CK). Red indicates increased transcript abundance relative to CK, blue indicates decreased transcript abundance, and white indicates a log_2_FC value close to zero. Gray cells indicate that expression was not detected or that a log_2_FC value could not be reliably estimated for the corresponding contrast. Genes are grouped according to their annotated functional categories, as indicated by the brackets on the left. APX, ascorbate peroxidase; DHAR, dehydroascorbate reductase; DP, dipeptidase involved in putative Cys–Gly hydrolysis; G6PD, glucose-6-phosphate dehydrogenase; GGT, γ-glutamyl transpeptidase; GPX, glutathione peroxidase; and GST, glutathione S-transferase. The heatmap represents transcript-level responses and does not indicate metabolite abundance, enzyme activity, pathway flux, or functional contribution to cold tolerance.

### 2.7 Statistical analysis

Physiological and qRT-PCR data were analyzed using SPSS Statistics version 27.0. The statistical test and multiple-comparison procedure used for each panel are specified in the corresponding figure legend; asterisks denote prespecified stage-matched cultivar comparisons in **Figure 3**, whereas letters denote *post hoc* groupings in **Figure 4**. Differential-expression and enrichment p-values were corrected by the Benjamini–Hochberg method as described above. TBtools-II (Chen et al., 2023) was used for sequence extraction and gene-structure inspection, and GraphPad Prism version 10.0 and OriginPro 2021 were used for visualization. For candidate-gene sequence comparisons, the complete CDS sequences extracted using TBtools-II were imported into DNAMAN for pairwise nucleotide sequence alignment.

**Figure 3:**
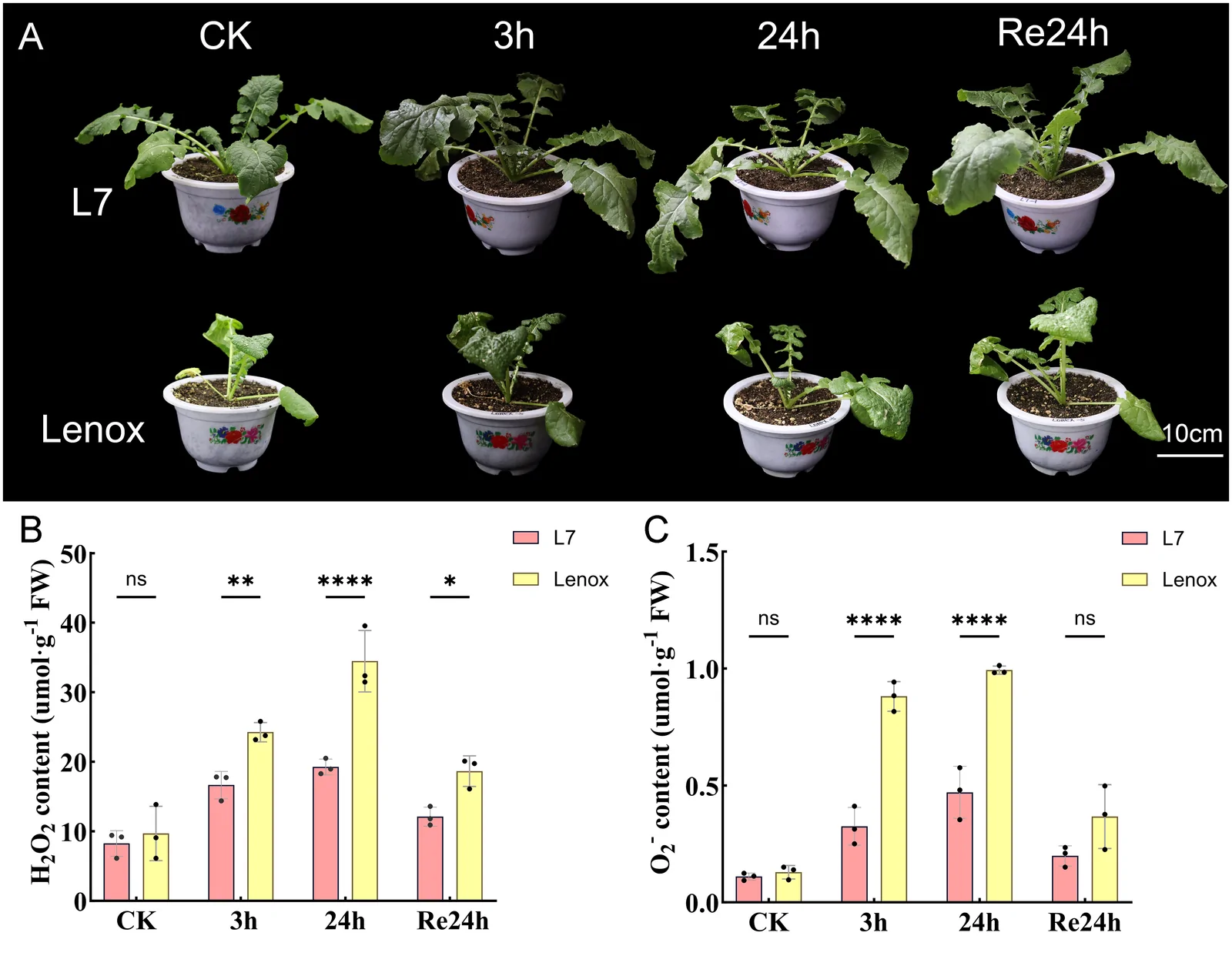
Phenotypic responses and reactive oxygen species accumulation in ‘Longyou-7’ and ‘Lenox’ during cold treatment and subsequent recovery. **(A)** Representative whole-plant phenotypes under CK conditions, after 3 and 24 h of treatment at 4 °C, and after 24 h of recovery at 22 °C following 24 h of cold treatment (Re24h). **(B)** H_2_O_2_ content and **(C)** O_2_^-^ content in growth-point tissues. Values are presented as the mean ± SD of three independent biological replicates. Asterisks indicate significant Differences between ‘Longyou-7’ and ‘Lenox’ at each sampling stage were assessed by two-way ANOVA followed by Šídák’s multiple comparisons test. Asterisks indicate the corresponding significance levels: *P* < 0.05, *P* < 0.01, *P* < 0.001, and *P* < 0.0001; ns, not significant.

**Figure 4:**
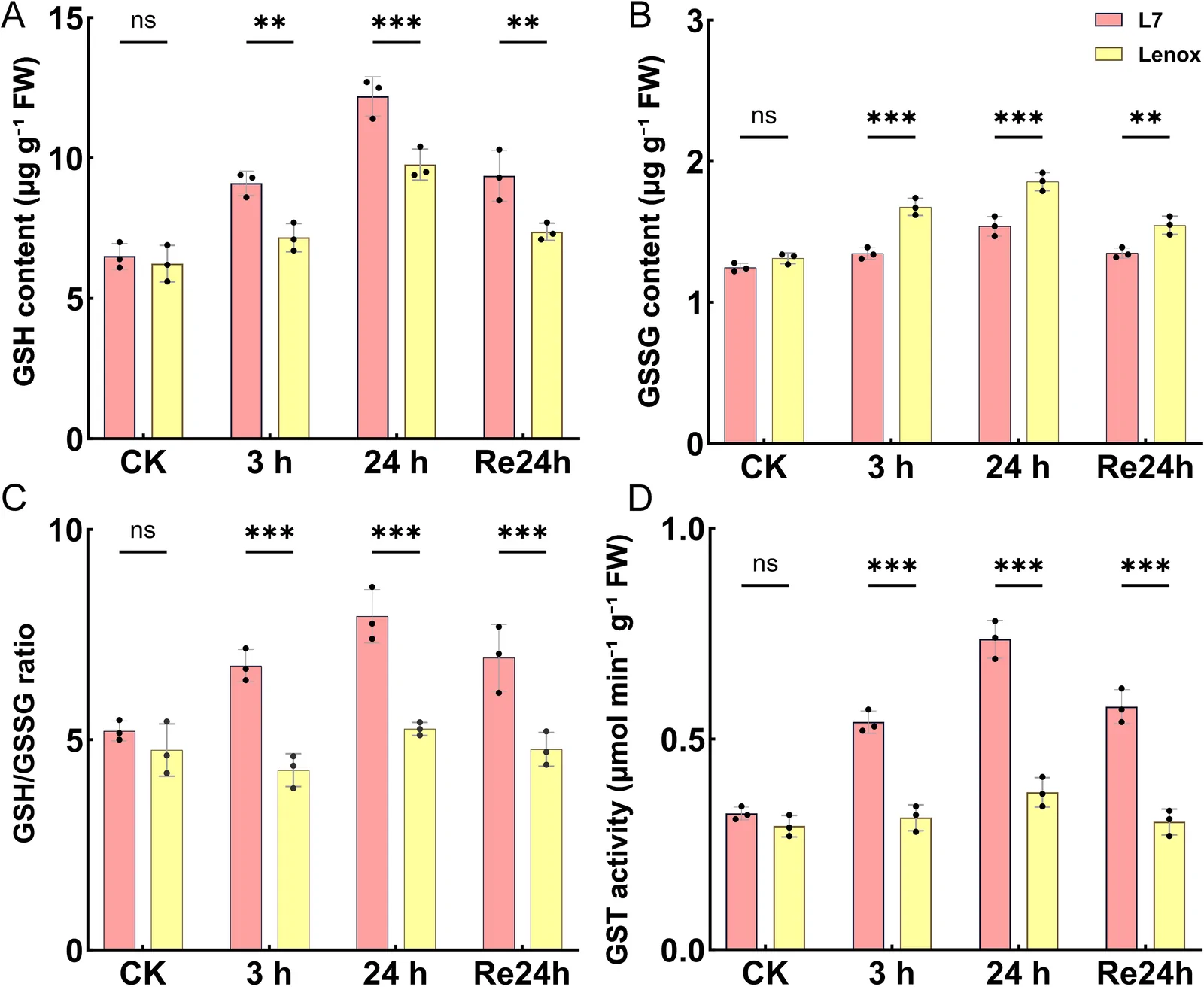
Biochemical responses of the glutathione system in ‘Longyou-7’ and ‘Lenox’ during cold treatment and recovery. **(A)** Reduced glutathione (GSH) content, **(B)** oxidized glutathione (GSSG) content, **(C)** GSH/GSSG ratio, **(D)** glutathione S-transferase (GST) activity under CK conditions, after 3 and 24 h of treatment at 4 °C, and after 24 h of recovery at 22 °C following 24 h of cold treatment (Re24h). The GSH/GSSG ratio was calculated separately for each biological replicate from the corresponding GSH and GSSG contents. Values are presented as the mean ± SD of three independent biological replicates, with individual biological replicate values shown as black points. Differences between ‘Longyou-7’ and ‘Lenox’ at each sampling stage were assessed by two-way ANOVA followed by Šídák’s multiple-comparisons test. Asterisks indicate the corresponding significance levels: P < 0.01 and P < 0.001; ns, not significant.

## 3 Results

### 3.1 Phenotypic observations and ROS accumulation during cold treatment

Representative whole-plant phenotypes of ‘Longyou-7’ and ‘Lenox’ were recorded under CK conditions, after 3 and 24 h of cold treatment, and following 24 h of recovery. After 24 h of cold treatment, ‘Lenox’ exhibited more pronounced leaf drooping and wilting, whereas the leaves of ‘Longyou-7’ remained relatively expanded. Following recovery, ‘Longyou-7’ showed visible phenotypic improvement, while ‘Lenox’ retained evident leaf curling and drooping (**Figure 3A**). In growth-point tissues, the mean H_2_O_2_ and O_2_^-^ contents increased during cold treatment and subsequently decreased during recovery in both cultivars (**Figures 3B, C**). Compared with ‘Longyou-7’, ‘Lenox’ exhibited significantly higher H_2_O_2_ contents at 3 h, 24 h, and Re24h, as well as significantly higher O_2_^-^ contents at 3 h and 24 h. These results indicate that ‘Lenox’ experienced greater ROS accumulation during cold treatment, whereas ‘Longyou-7’ maintained a comparatively lower level of oxidative stress.

### 3.2 Transcriptomic overview and reference-genome comparison

Principal component analysis showed clear separation of the RNA-seq samples by cultivar and treatment, with PC1 and PC2 explaining 36.23% and 17.60% of the total variance, respectively, and biological replicates clustering closely (**Figure 5A**). Within-genotype comparisons relative to the corresponding CK identified 1,465, 6,922, and 1,684 DEGs in ‘Longyou-7’ at 3 h, 24 h, and Re24h, respectively, and 2,832, 5,646, and 2,231 DEGs in ‘Lenox’ at the corresponding stages (**Figure 5C**). Among these CK-relative responses, 256 DEGs in ‘Longyou-7’ and 563 DEGs in ‘Lenox’ were shared across all three treatment stages (**Figures 5B, D**). The direct recovery comparison, Re24h versus 24 h, identified 7,149 DEGs in ‘Longyou-7’ and 5,013 DEGs in ‘Lenox’ (**Figure 5C**). Comparison of the two reference annotations linked 40,492 Longyou-7 gene models to 34,486 Chiifu v1.5 genes, whereas 5,352 Longyou-7 gene models and 6,532 Chiifu v1.5 genes were outside the retained matched set (**Figure 5E**). The Longyou-7 reference increased uniquely mapped read rates in Longyou-7 samples by 1.28–1.69 percentage points relative to Chiifu v1.5, whereas differences in ‘Lenox’ ranged from −0.26 to 0.30 percentage points (**Figure 5F**). In the harmonized reference-genome comparison of the six CK-relative within-genotype contrasts, shared best-hit DEG pairs showed 99.41–99.93% directional concordance and Pearson correlations of 0.967–0.994 for log_2_ fold changes, while DEG-group Jaccard indices ranged from 0.390 to 0.604 (**Supplementary Table 1**). Longyou-7-reference DEGs without a retained Chiifu v1.5 BLAST hit accounted for 2.06–3.34% of the corresponding DEG sets (**Supplementary Tables 1**, **2**).

**Figure 5:**
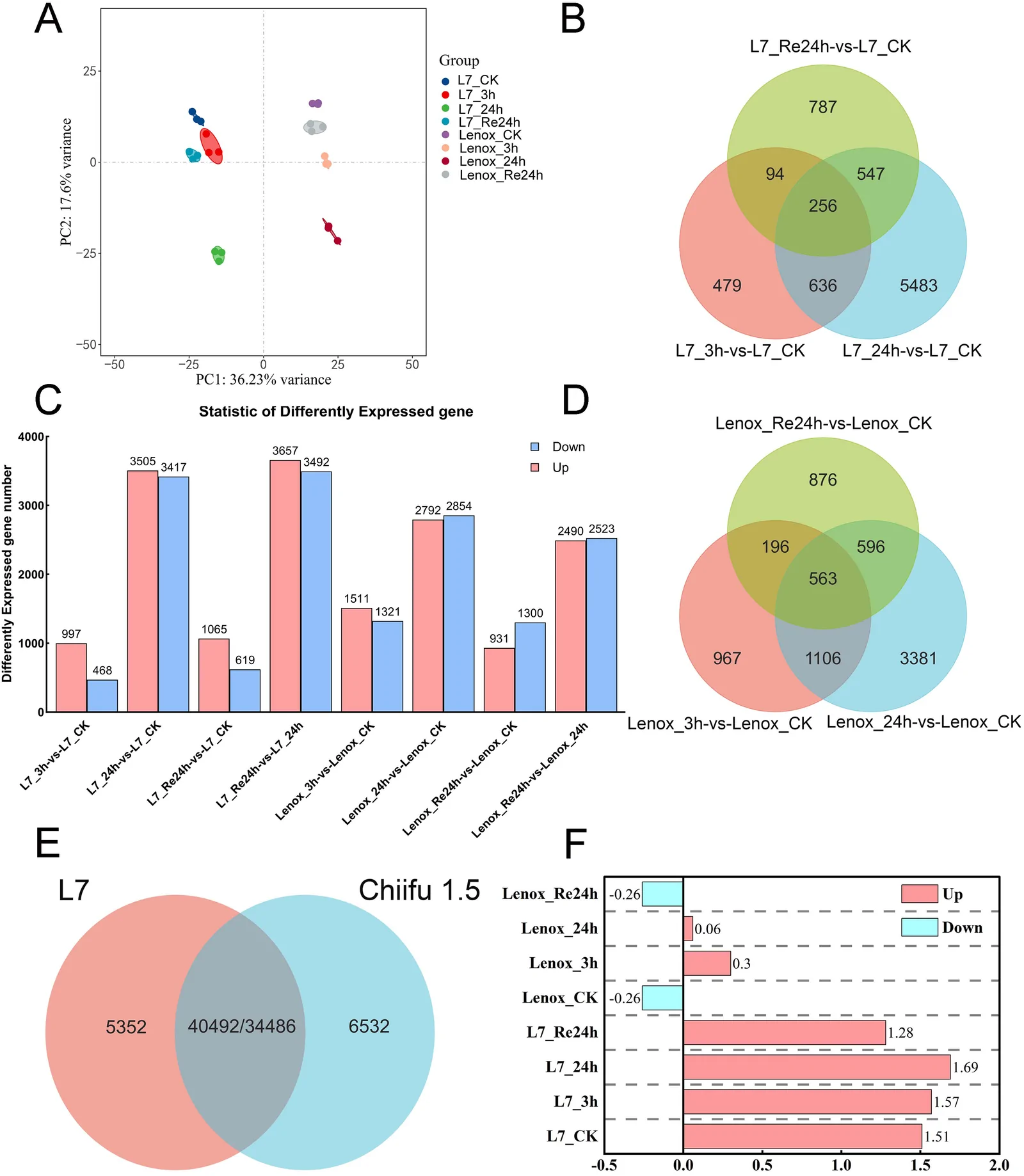
Transcriptomic overview of within-genotype responses during cold treatment and recovery and comparison of the Longyou-7 and Chiifu v1.5 reference genomes. **(A)** Principal component analysis of the 24 RNA-seq samples from ‘Longyou-7’ and ‘Lenox’ at CK, 3 h, 24 h, and Re24h. **(B)** Venn diagram showing the overlap among DEGs identified in ‘Longyou-7’ at 3 h, 24 h, and Re24h relative to the corresponding CK. **(C)** Numbers of upregulated (Up) and downregulated (Down) DEGs in the within-genotype contrasts of 3 h versus CK, 24 h versus CK, Re24h versus CK, and Re24h versus 24 h for ‘Longyou-7’ and ‘Lenox’. Up and Down indicate higher and lower transcript abundance, respectively, in the first condition relative to the second condition in each contrast. **(D)** Venn diagram showing the overlap among DEGs identified in ‘Lenox’ at 3 h, 24 h, and Re24h relative to the corresponding CK. **(E)** Correspondence between the annotated gene sets of the Longyou-7 and Chiifu v1.5 reference genomes. The values 40,492/34,486 in the shared region indicate 40,492 Longyou-7 gene models with retained matches and 34,486 corresponding Chiifu v1.5 genes, respectively. **(F)** Percentage-point differences in uniquely mapped read rates obtained using the Longyou-7 and Chiifu v1.5 reference genomes across the eight cultivar-by-stage sample groups. Positive values indicate higher uniquely mapped read rates with the Longyou-7 reference, whereas negative values indicate higher rates with Chiifu v1.5. DEGs in panels B–D were defined using a Benjamini–Hochberg-adjusted P < 0.05 and |log_2_FC| ≥ 1.

### 3.3 Functional enrichment during cold treatment and recovery

GO and KEGG enrichment analyses of the stage-matched Longyou-7-versus-Lenox DEGs showed marked changes in functional profiles during cold treatment. At 3 h, the enriched GO terms were mainly related to chromatin and transcriptional regulation, including negative regulation of histone H3K27 methylation and cell differentiation, while glutathione metabolism was represented in the KEGG analysis (**Figures 6A, C**). By 24 h, glutathione-related functions became more prominent, with glutathione metabolic process and glutathione transferase activity among the enriched GO terms and glutathione metabolism represented in the KEGG pathways (**Figures 6B, D**). Glutathione metabolism-related genes also showed distinct expression patterns across CK, 3 h, 24 h, and Re24h under both the Longyou-7 and Chiifu v1.5 annotation backgrounds (**Figures 6E, F**). At CK, the between-cultivar differences were mainly associated with basal metabolic and cellular processes, whereas the Re24h comparison included hormone- and signaling-related functions such as gibberellin response, abscisic acid binding, plant hormone signal transduction, and MAPK signaling (**Supplementary Figure 1**).Within-genotype analyses further revealed stage-dependent changes relative to the corresponding CK (**Supplementary Figure 2**). Photosynthesis, circadian regulation, hormone signaling, lipid-related processes, and primary and secondary metabolism were represented to different extents across the two cultivars and treatment stages. Glutathione metabolism was among the top-ranked pathways at 3 h, 24 h, and Re24h in ‘Longyou-7’, and at 24 h and Re24h in ‘Lenox’. During recovery, comparison of Re24h with 24 h showed that photosynthesis and hormone signaling were represented in both cultivars, together with MAPK, peroxisomal, lipid, and amino acid-related processes in ‘Longyou-7’ and sulfur, alpha-linolenic acid, and amino acid-related processes in ‘Lenox’ (**Supplementary Figure 3**).

**Figure 6:**
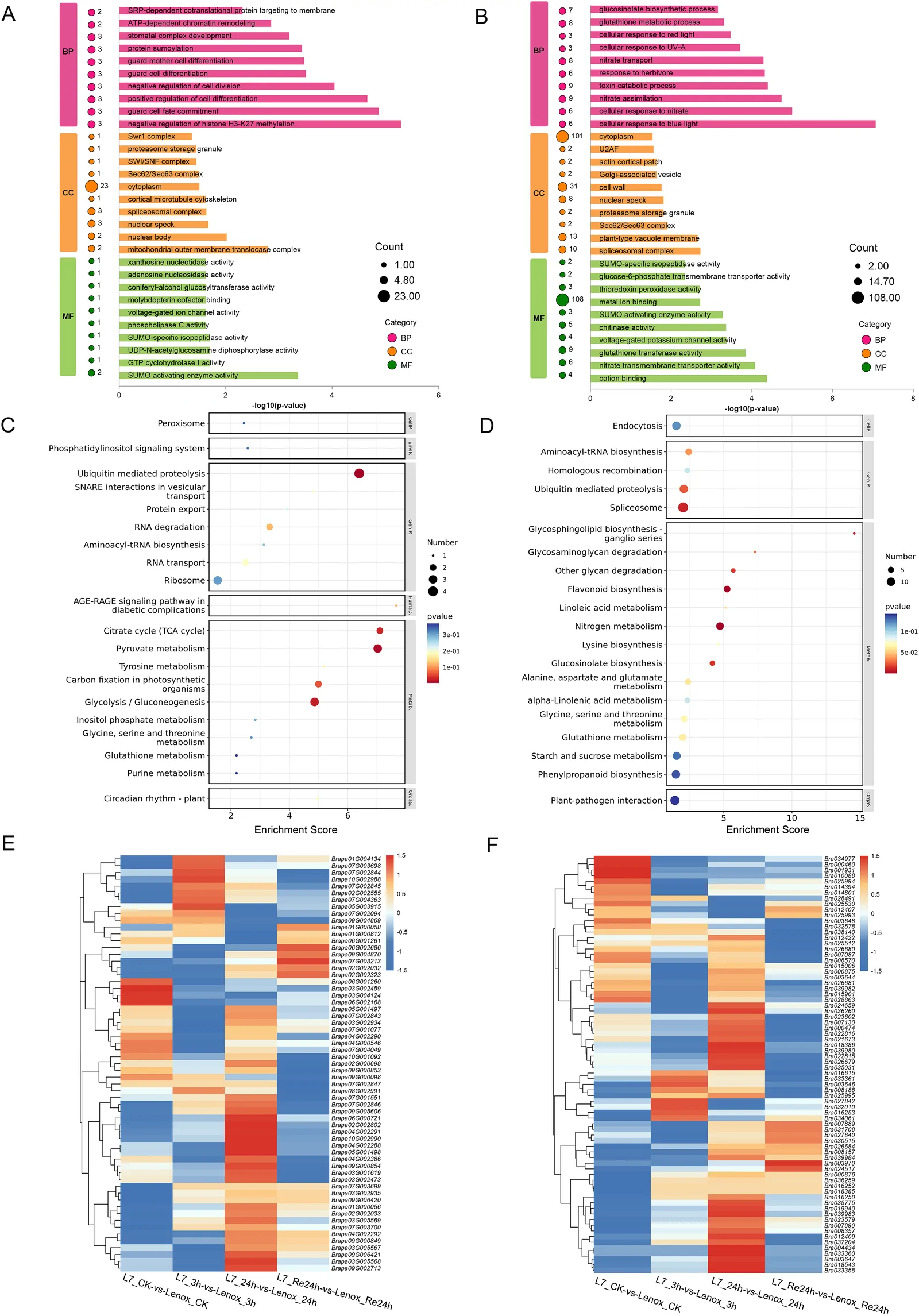
GO and KEGG enrichment analyses of Longyou-7-versus-Lenox DEGs and expression patterns of glutathione metabolism-related genes. **(A, B)** GO enrichment analyses of DEGs identified at 3 h **(A)** and 24 h **(B)**, grouped into biological process (BP), cellular component (CC), and molecular function (MF) categories. Bar length represents −log_10_(P-value), and the numbers beside the terms indicate the corresponding gene counts. **(C, D)** KEGG enrichment analyses of DEGs identified at 3 h **(C)** and 24 h **(D)**. Bubble size represents the number of genes assigned to each pathway, bubble color represents the enrichment P-value, and the horizontal axis indicates the enrichment score. **(E, F)** Hierarchically clustered heatmaps showing the relative expression patterns of glutathione metabolism-related genes identified using the Longyou-7 reference genome **(E)** and the Chiifu v1.5 reference genome **(F)**. Columns correspond to the CK, 3 h, 24 h, and Re24h stages. Red indicates a relatively high expression level, blue indicates a relatively low expression level, and colors close to white indicate an intermediate expression level. The numerical range of the color scale is shown beside each heatmap.

### 3.4 Expression-pattern clustering and functional enrichment

To summarize the expression profiles of DEGs across the eight cultivar-by-stage groups, genes identified from the within-genotype and genotype-by-stage interaction analyses were subjected to expression-pattern clustering. The DEGs were assigned to 17 expression-pattern clusters (**Figure 7A**). Clusters a and e showed reduced expression during cold treatment followed by increased expression during recovery (**Supplementary Figures 4A, E**), indicating cold-repressed and recovery-associated profiles. By contrast, clusters i and j displayed transient increases at 3 h followed by lower expression at 24 h or Re24h, although the magnitude of these changes differed between the two cultivars (**Figure 7B**). Functional enrichment analysis showed that genes in clusters a and e were mainly associated with peroxisomes, fatty acid β-oxidation, lipid metabolism, and ethylene-activated signaling (**Supplementary Figures 5A–D**). Genes in clusters i and j were enriched in sulfur amino acid metabolism, cysteine biosynthesis, and related processes (**Figures 8A–D**). Because cysteine is a precursor of GSH biosynthesis, these enrichment patterns were consistent with the transcript-level involvement of GSH- and GST-related processes. The GST-related candidate *Brapa09G004869* was assigned to cluster i (**Figure 8E**), which displayed a transient increase at 3 h followed by lower expression at later stages.

**Figure 7:**
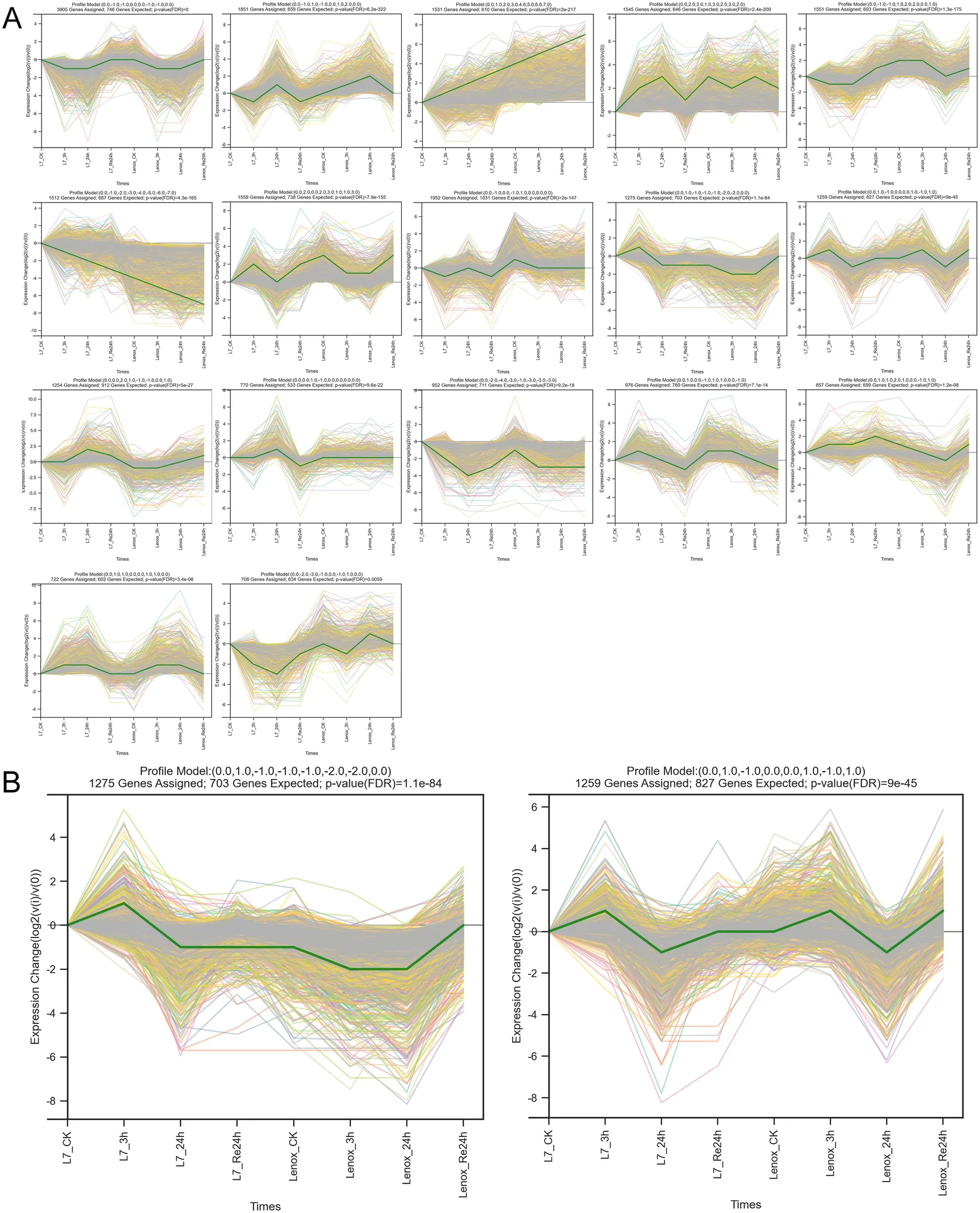
Expression-pattern clustering of DEGs across the eight cultivar-by-stage groups. **(A)** Seventeen DEG expression-pattern clusters across L7_CK, L7_3h, L7_24h, L7_Re24h, Lenox_CK, Lenox_3h, Lenox_24h, and Lenox_Re24h. Individual lines represent the expression profiles of individual genes, and the green line represents the model expression profile of each cluster. **(B)** Enlarged expression profiles of representative clusters i and **(i)** The two cultivars constitute separate treatment series and are displayed together to facilitate comparison of their expression patterns.

**Figure 8:**
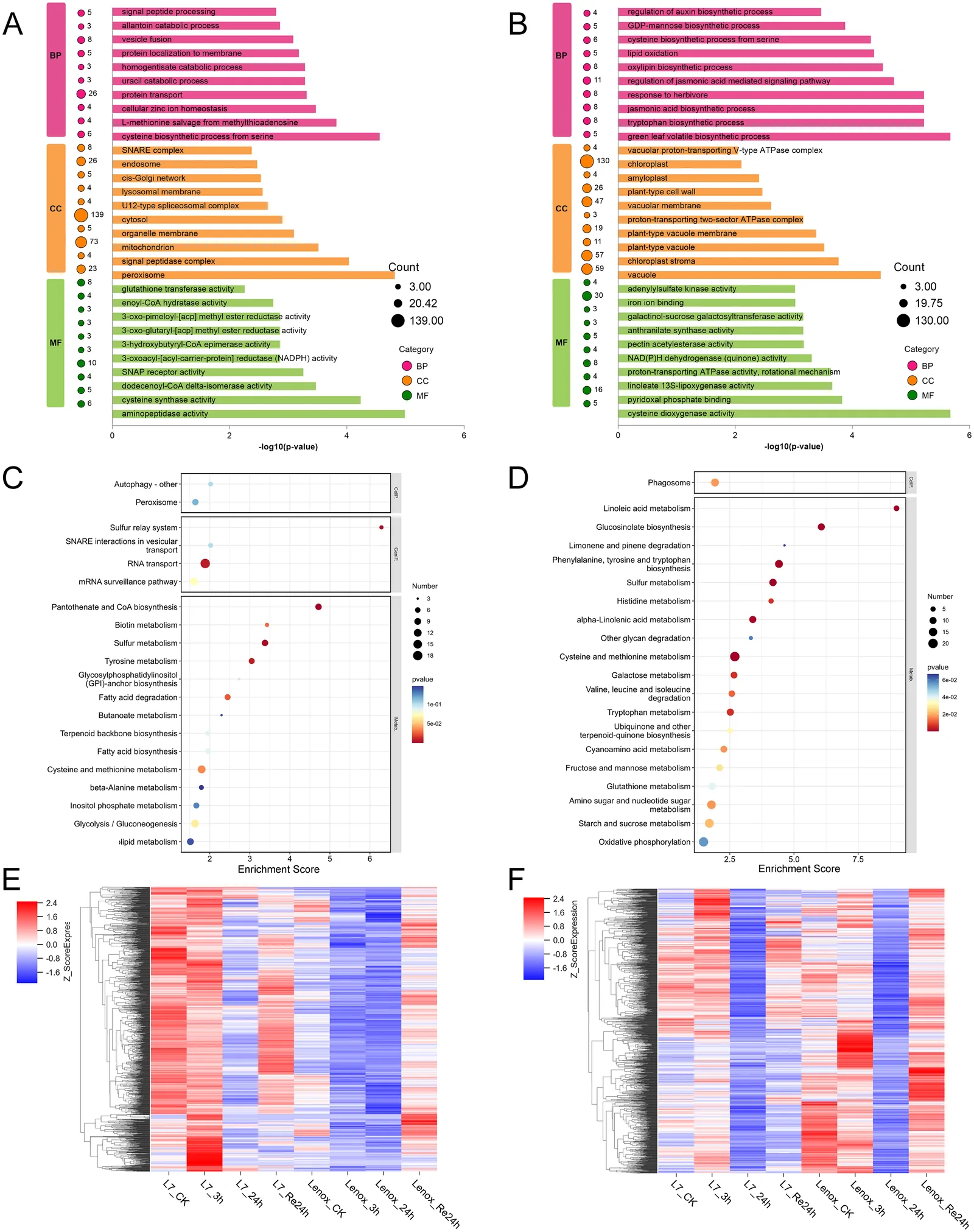
Functional enrichment analyses and expression heatmaps of representative expression-pattern clusters. **(A, B)** GO enrichment analyses of genes in cluster i **(A)** and cluster j **(B)**, grouped into biological process (BP), cellular component (CC), and molecular function (MF) categories. Bar length represents −log_10_(P-value), and the numbers and symbol sizes indicate the numbers of genes assigned to the corresponding terms. **(C, D)** KEGG enrichment analyses of genes in cluster i **(C)** and cluster j **(D)**. The horizontal axis represents the enrichment score, bubble size represents the number of genes assigned to each pathway, and bubble color indicates the enrichment P-value. **(E, F)** Heatmaps of standardized expression levels for genes in cluster i **(E)** and cluster j **(F)** across the eight cultivar-by-stage groups. Genes are shown in rows and were hierarchically clustered, whereas columns represent L7_CK, L7_3h, L7_24h, L7_Re24h, Lenox_CK, Lenox_3h, Lenox_24h, and Lenox_Re24h. Red indicates relatively high expression, blue indicates relatively low expression, and white indicates expression near the gene-specific mean. Expression values were standardized by gene using row-wise Z-score transformation.

### 3.5 Genotype-specific WGCNA and module–trait associations

Weighted gene co-expression network analysis was performed separately for ‘Longyou-7’ and ‘Lenox’ to characterize within-genotype patterns of gene co-expression during cold treatment and recovery. The ‘Longyou-7’ network initially contained 61 dynamic modules, which were merged into 24 modules, whereas the ‘Lenox’ network contained 113 initial modules that were merged into 25 modules (**Figures 9A, B**). Module colors were assigned independently in the two networks and therefore do not represent corresponding modules between the cultivars. Module–trait analysis identified associations between module eigengenes and POD, CAT, soluble sugar, MDA, H_2_O_2_, and O_2_^-^ within each genotype (**Figures 9C, D**). In ‘Longyou-7’, the darkslateblue and coral1 modules contained genes annotated to glutathione metabolism and showed associations with antioxidant- and ROS-related physiological traits. Simplified hub-gene co-expression subnetworks were further generated for these two modules, highlighting the top 10 hub genes and their mutual co-expression relationships (**Supplementary Figure 10**). Other modules also exhibited positive or negative associations with individual physiological measurements. In ‘Lenox’, glutathione metabolism-related genes were distributed among independently defined modules that showed their own patterns of association with the measured physiological traits. Together, the genotype-specific networks revealed distinct patterns of co-variation among glutathione-related transcription, antioxidant enzyme activities, and ROS-associated physiological traits.

**Figure 9:**
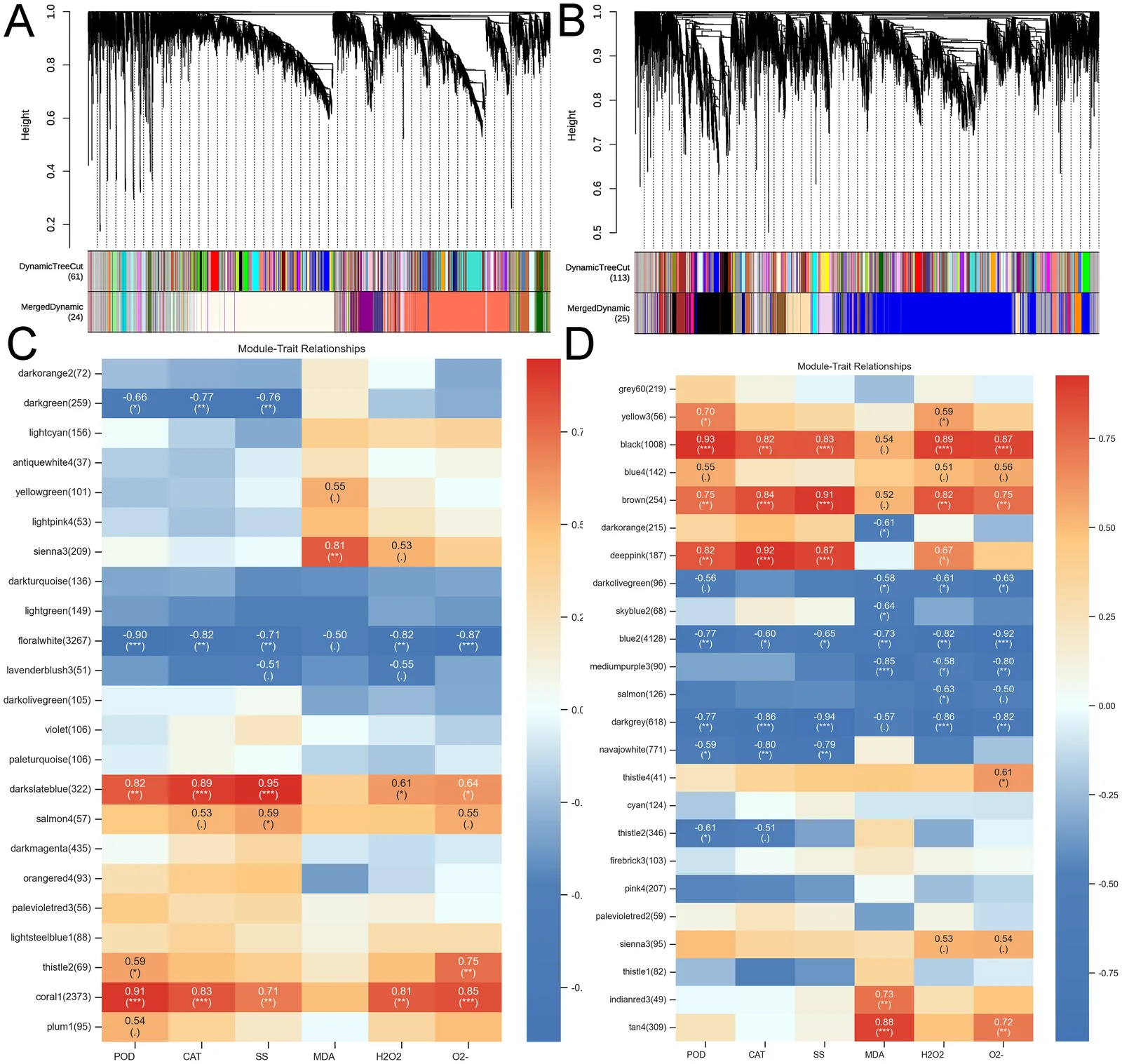
Genotype-specific WGCNA network construction and module–trait relationships in ‘Longyou-7’ and ‘Lenox’.**(A)** Gene clustering dendrogram and module assignment for ‘Longyou-7’. The upper color bar shows the 61 initial modules identified using dynamic tree cutting, and the lower color bar shows the 24 modules obtained after merging modules with similar eigengene profiles. **(B)** Gene clustering dendrogram and module assignment for ‘Lenox’. The upper and lower color bars represent the 113 initial modules and the 25 merged modules, respectively. **(C, D)** Correlations between module eigengenes and the physiological traits POD, CAT, soluble sugar content (SS), MDA, H_2_O_2_, and O_2_^-^ in ‘Longyou-7’ **(C)** and ‘Lenox’ **(D)**, respectively. Each correlation analysis was performed using the 12 samples of the corresponding genotype across CK, 3 h, 24 h, and Re24h. Rows represent co-expression modules, with the number of genes in each module shown in parentheses, and columns represent physiological traits. Cell values indicate Pearson correlation coefficients, with red and blue representing positive and negative correlations, respectively. Asterisks indicate correlation significance: P < 0.05, P < 0.01, and P < 0.001. Module colors were assigned independently in the two genotype-specific networks and therefore do not indicate direct correspondence between ‘Longyou-7’ and ‘Lenox’.

### 3.6 Annotation-based mapping and expression patterns of glutathione metabolism-related genes

Based on functional annotations, 39 glutathione metabolism-related genes were positioned within an integrated glutathione-associated metabolic framework (**Figure 1**). The reconstructed scheme comprised several interconnected modules, including the ascorbate–glutathione (AsA–GSH) cycle, glutathione biosynthesis, glutathione peroxidase-associated peroxide reduction, pentose phosphate-associated NADPH generation, GST-mediated glutathione conjugation, and glutathione degradation. Within the AsA–GSH cycle, APX-, DHAR-, and GR-associated reactions linked H_2_O_2_ reduction with GSH/GSSG recycling, whereas GSH1- and GSH2-associated reactions represented the two-step biosynthetic route from glutamate, cysteine, and glycine to GSH. G6PD was positioned as a potential source of NADPH for reductive recycling, while GPX- and GST-associated reactions represented GSH-consuming branches related to peroxide reduction and conjugation, respectively. GGT- and dipeptidase-associated reactions were assigned to the glutathione degradation and γ-glutamyl-cycle-related branch. The transcript-level responses of these genes were then examined using the six within-genotype contrasts: L7_3h/CK, L7_24h/CK, L7_Re24h/CK, Lenox_3h/CK, Lenox_24h/CK, and Lenox_Re24h/CK (**Figure 2**). The 39 genes displayed heterogeneous and stage-dependent expression patterns across the two cultivars. Genes assigned to the GST group constituted the largest portion of the mapped set and showed both induction and repression depending on genotype and treatment stage. Distinct responses were also observed among genes associated with the AsA–GSH cycle, GPX-mediated peroxide reduction, NADPH generation, and glutathione turnover. These patterns indicate that the glutathione-associated transcriptional response involved multiple components of the network rather than a uniform change in a single metabolic branch. Full-length CDS comparisons were additionally performed for the GST-related genes *Brapa09G004869* and *Brapa10G002988* and their best-matching Chiifu v1.5 sequences. Pairwise nucleotide identities were 83.51% and 74.56%, respectively (**Supplementary Figure 9**).

### 3.7 Biochemical responses of the glutathione system during cold treatment and recovery

To determine whether the transcriptional changes associated with glutathione metabolism were accompanied by biochemical variation, GSH and GSSG contents, the GSH/GSSG ratio, and GST activity were examined in growth-point tissues across CK, 3 h, 24 h, and Re24h (**Figure 4**). Under CK conditions, none of the four measurements differed significantly between ‘Longyou-7’ and ‘Lenox’. During cold treatment, GSH content increased in both cultivars and reached its highest mean level at 24 h. Compared with ‘Lenox’, ‘Longyou-7’ showed higher GSH contents at 3 h (P < 0.01), 24 h (P < 0.001), and Re24h (P < 0.01). In contrast, GSSG accumulated to higher levels in ‘Lenox’, with significant cultivar differences at 3 h and 24 h (P < 0.001) and at Re24h (P < 0.01). Consequently, the GSH/GSSG ratio was higher in ‘Longyou-7’ than in ‘Lenox’ at 3 h, 24 h, and Re24h (all P < 0.001). GST activity also showed a pronounced increase in ‘Longyou-7’ during cold treatment, reaching its highest mean level at 24 h, whereas the corresponding increase in ‘Lenox’ was comparatively smaller. GST activity was significantly higher in ‘Longyou-7’ than in ‘Lenox’ at 3 h, 24 h, and Re24h. Following 24 h of recovery, GSH content, GSSG content, and GST activity decreased from their 24-h levels in both cultivars, while the higher GSH/GSSG ratio in ‘Longyou-7’ was maintained. Together, these biochemical profiles accompanied the stage-dependent transcriptional changes observed for glutathione metabolism-related genes during cold treatment and recovery.

### 3.8 qRT-PCR assessment of candidate gene expression

qRT-PCR analysis was performed for 22 glutathione metabolism-related candidate genes, comprising 15 high-abundance candidates, six low-abundance candidates, and one gene uniquely annotated in the Longyou-7 reference genome. Eight of the 15 high-abundance candidates overlapped with the 39 glutathione metabolism-related genes included in **Figures 1** and **2**. The representative set comprised the Longyou-7-specific annotated gene *Brapa01G000257*, three low-abundance candidates (*Brapa02G002033*, *Brapa09G000849*, and *Brapa10G001092*), and four high-abundance candidates (*Brapa02G000698*, *Brapa09G004869*, *Brapa09G006420*, and *Brapa10G002988*) (**Figure 10**). The other 14 validated genes showed corresponding expression profiles across the cultivar-by-stage combinations (**Supplementary Figure 6**). Across the two cultivars and four treatment stages, qRT-PCR and RNA-seq showed generally consistent expression trajectories for the validated genes, including transient and non-monotonic changes during cold treatment and recovery. Linear regression analysis based on the mean log_2_ fold-change values of all 22 validated genes showed a positive relationship between the RNA-seq and qRT-PCR measurements (R² = 0.736; **Figure 10I**), indicating overall concordance between the two transcript-level expression datasets. Seventeen qRT-PCR candidates contained the characteristic GST_N and GST_C domains; class-specific CDD assignments classified nine as Tau GSTs and eight as Phi GSTs. Phylogenetic analysis further revealed conserved motif patterns and domain architectures among these candidates (**Supplementary Figures 7**, **8**).

**Figure 10:**
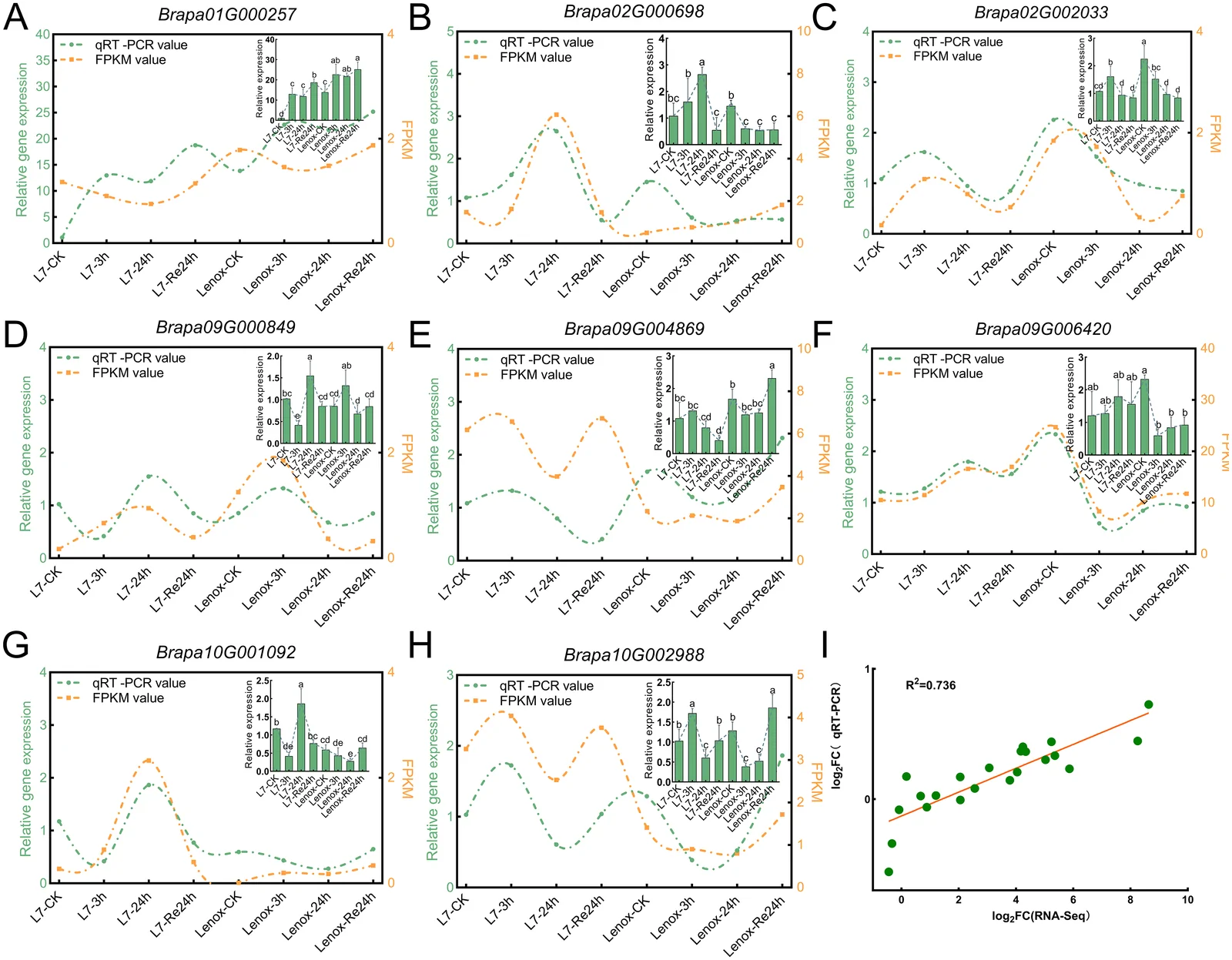
qRT-PCR assessment of representative glutathione metabolism-related candidate genes and concordance with RNA-seq. **(A–H)** Expression profiles of eight representative candidate genes across CK, 3 h, 24 h, and Re24h in ‘Longyou-7’ and ‘Lenox’. The eight genes comprise one Longyou-7-specific annotated candidate (*Brapa01G000257*), three low-abundance candidates (*Brapa02G002033*, *Brapa09G000849*, and *Brapa10G001092*), and four high-abundance candidates (*Brapa02G000698*, *Brapa09G004869*, *Brapa09G006420*, and *Brapa10G002988*). Green dashed lines indicate qRT-PCR relative expression profiles, whereas orange dashed lines indicate RNA-seq FPKM values. Insets show qRT-PCR relative expression across the eight cultivar-by-stage groups, with values presented as the mean ± SD of three independent biological replicates. Different lowercase letters indicate significant differences among the eight groups based on one-way ANOVA followed by Duncan’s multiple range test (*P* < 0.05); groups sharing at least one letter are not significantly different. **(I)** Linear regression between RNA-seq and qRT-PCR mean log_2_ fold-change values for all 22 validated genes. Each point represents one gene (R² = 0.736).

## 4 Discussion

### 4.1 Reference-genome choice mainly affects gene-level detection

Reference genome and annotation quality can influence read alignment, transcript quantification, and differential gene detection, particularly when the analyzed genotype differs genetically from the reference background (Slabaugh et al., 2019; Guo et al., 2022). In the present study, use of the Longyou-7 reference increased uniquely mapped read rates in Longyou-7 samples by 1.28–1.69 percentage points relative to Chiifu v1.5, whereas little change was observed in ‘Lenox’ (−0.26 to 0.30 percentage points). In the harmonized comparison across the six CK-relative within-genotype contrasts, shared best-hit DEG pairs showed very high directional concordance (99.41–99.93%) and closely correlated log_2_ fold changes (*r* = 0.967–0.994), although DEG-group overlap varied more substantially among contrasts (Jaccard index = 0.390–0.604). These results indicate that the genotype-matched reference modestly improved read representation and influenced which genes were detected as differentially expressed, while the direction and magnitude of shared DEG responses remained highly consistent between the two reference backgrounds. The effect of reference choice was therefore more apparent at the level of individual gene detection and annotation than in the overall transcriptional response. Longyou-7-reference DEGs without a retained Chiifu v1.5 BLAST hit accounted for only 2.06–3.34% of the corresponding DEG sets, and among the evaluated no-hit subsets, FDR-significant pathway enrichment was sparse. Such no-hit genes may reflect sequence divergence, differences in gene models, or annotation completeness and should not be interpreted as evidence of genomic absence from Chiifu v1.5. Differences between the present analysis and the previous Chiifu v1.5-based study therefore cannot be attributed solely to reference-genome choice because the two analyses also differed in statistical contrasts, DEG definitions, filtering procedures, annotation backgrounds, and enrichment strategies. The glutathione-, phenylpropanoid-, and carbohydrate-related processes emphasized in the two analyses are better viewed as complementary features of the winter-rapeseed cold-response transcriptome revealed under different analytical frameworks.

### 4.2 Temporal reprogramming during cold treatment and recovery

Plant acclimation to low temperature involves coordinated changes in signaling, photosynthesis, membrane properties, metabolism, and antioxidant processes (Waititu et al., 2021; Tian et al., 2022). In the present study, transcriptional responses varied markedly across treatment stages. At 3 h, the stage-matched comparison highlighted chromatin-related regulation, whereas the within-genotype profiles emphasized photosynthesis, fatty acid elongation, and secondary metabolism in ‘Longyou-7’ and hormone signaling, alpha-linolenic acid metabolism, circadian regulation, and amino acid-related pathways in ‘Lenox’. By 24 h, ribosome biogenesis, circadian regulation, signaling, and broader metabolic processes became prominent, indicating a shift from early regulatory adjustment to more extensive transcriptional reprogramming during prolonged cold exposure. The two cultivars were sampled at the same chronological age and under the same treatment schedule but were not matched for morphological developmental stage, and their CK phenotypes already showed visible differences in plant size and overall development (**Figure 3A**). Accordingly, direct stage-matched cultivar comparisons may reflect both constitutive genetic differences and variation associated with developmental status and were therefore interpreted together with the within-genotype and genotype × stage analyses. Recovery represented a further transition. Relative to 24 h, recovery-upregulated genes were associated with photosynthesis and hormone signaling in both cultivars, together with MAPK, peroxisomal, lipid, and amino acid-related processes in ‘Longyou-7’ and sulfur, alpha-linolenic acid, and amino acid-related processes in ‘Lenox’. These changes are consistent with active transcriptional readjustment after plants returned to 22 °C and may be relevant to the fluctuating temperatures experienced by winter rapeseed in the field.

### 4.3 Gene expression patterns and their functional associations across treatment conditions

Expression-pattern clustering revealed distinct temporal profiles across cold treatment and recovery. Clusters a and e showed reduced expression during cold exposure followed by recovery-associated increases, whereas clusters i and j displayed an early increase followed by lower expression at later stages. The functional associations of these clusters further suggest that cold-responsive transcription involved coordinated changes in peroxisomal functions, fatty acid β-oxidation, lipid metabolism, ethylene signaling, and sulfur amino acid metabolism. Lipid remodeling and peroxisomal metabolism are closely associated with membrane adjustment and oxidative metabolism during cold stress (Waititu et al., 2021), while the enrichment of cysteine-related processes in the early-response clusters is relevant to glutathione metabolism because cysteine is a precursor for GSH biosynthesis (Liu, 2022; Wang et al., 2024). The GST-related candidate *Brapa09G004869* was also assigned to the early-response cluster i, further linking GSH/GST-related transcription with this transient expression pattern. Together, these patterns place glutathione-related genes within a broader, stage-dependent metabolic adjustment rather than indicating a uniform response throughout cold treatment and recovery.

### 4.4 Co-expression modules associated with antioxidant and osmotic adjustment traits

Cold-induced ROS accumulation is usually accompanied by changes in antioxidant activity and osmotic adjustment, which together contribute to the maintenance of cellular homeostasis under low-temperature stress. In the present study, CAT and POD activities increased together with H_2_O_2_ and O_2_^-^ in several module–trait associations. Rather than indicating a simple increase in ROS-scavenging capacity, this parallel variation may reflect the induction of antioxidant defenses in response to increasing oxidative stress. Similar increases in antioxidant enzyme activities during cold exposure have been widely reported as part of the physiological adjustment to ROS accumulation. The WGCNA results further connected these physiological changes with transcriptional variation. In ‘Longyou-7’, modules containing glutathione metabolism-related genes were associated with CAT, POD, soluble sugar, H_2_O_2_, and O_2_^-^, suggesting that changes in redox-related transcription occurred together with antioxidant and osmotic responses during cold treatment and recovery. The simplified hub-gene subnetworks of the coral1 and darkslateblue modules further illustrated highly connected transcriptional components within these glutathione-associated modules. ‘Lenox’ also showed associations between co-expression modules and antioxidant- or ROS-related traits, although the genes represented in these modules and their correlation patterns were different. Taken together, these results suggest that transcriptional responses associated with glutathione metabolism formed part of a broader adjustment involving ROS accumulation, antioxidant activity, and osmotic regulation in both cultivars.

### 4.5 Glutathione-related transcriptional and biochemical responses during cold treatment and recovery

Cold stress is often accompanied by ROS accumulation, and glutathione metabolism contributes to cellular redox homeostasis (Hasanuzzaman et al., 2020; Wang et al., 2024). In the present study, glutathione metabolism was among the top-ranked pathways at several treatment stages in both cultivars, and the 39 glutathione metabolism-related genes represented multiple components of this pathway, including the AsA–GSH cycle, glutathione biosynthesis and turnover, peroxide reduction, and GST-mediated conjugation. These transcriptional changes were accompanied by corresponding biochemical variation during cold treatment and recovery. In particular, GSH content and GST activity increased during cold exposure, while ‘Longyou-7’ maintained a higher GSH/GSSG ratio and lower GSSG accumulation than ‘Lenox’ at the cold-treatment stages. These patterns indicate that glutathione-related transcriptional adjustment occurred together with changes in glutathione redox status and GST activity during the cold response. The qRT-PCR results further supported the RNA-seq expression patterns across candidates with different transcript abundances. In addition, 17 candidates contained conserved GST_N and GST_C domains and were assigned to the Tau or Phi subclasses, providing sequence-based support for their annotation as cytosolic GSTs. Comparative analyses with GST-related proteins from Chiifu, *Arabidopsis thaliana*, and *Brassica napus* further placed these candidates within a broader phylogenetic context. Together with the antioxidant-, peroxisome-, phenylpropanoid-, and carbohydrate-related responses observed in the present and previous analyses of these cultivars (Ma et al., 2019), the biochemical and transcriptional data place GSH/GST-related processes within a broader network of metabolic and oxidative adjustment during cold treatment and recovery. However, these associations do not by themselves establish that altered glutathione metabolism or GST activity causally determines the contrasting cold tolerance of the two cultivars. Functional analyses of individual candidate genes will therefore be required to define their specific contributions to cold tolerance.

## 5 Conclusions

This study provides a reference-aware view of transcriptional changes in winter rapeseed during cold treatment and subsequent recovery. Within-genotype analyses revealed marked stage-dependent transcriptional responses in both ‘Longyou-7’ and ‘Lenox’, involving photosynthetic, lipid-related, hormone-signaling, circadian, sulfur and amino acid metabolism, peroxisomal, and other stress-associated processes. The transition from 24 h of cold treatment to Re24h was accompanied by extensive transcriptional readjustment, further indicating that recovery represents a distinct phase of the response rather than a simple return to the control state. Genotype-specific co-expression networks further linked transcriptional variation with antioxidant enzyme activities, soluble sugar, and ROS-associated traits. Glutathione metabolism was represented among the top-ranked pathways in several within-genotype contrasts, and the 39 glutathione metabolism-related genes showed heterogeneous expression patterns across cold treatment and recovery. Biochemical measurements further showed coordinated changes in GSH and GSSG contents, the GSH/GSSG ratio, and GST activity. During cold treatment, ‘Longyou-7’ maintained a higher GSH/GSSG ratio and higher GST activity than ‘Lenox’, providing biochemical support for the involvement of glutathione-related processes in the cold response. qRT-PCR assessment of 22 candidates generally supported the RNA-seq expression trends, while conserved-domain analysis classified 17 GST candidates into nine Tau and eight Phi GSTs. Together, the transcriptional and biochemical results support GSH/GST-related processes as one component of the broader cold-response network and identify GST-related genes as candidates for subsequent functional and genetic investigation, without establishing a GSH-centered causal mechanism of cold tolerance. Comparison of the Longyou-7 and Chiifu v1.5 reference backgrounds showed a modest improvement in uniquely mapped reads for Longyou-7 samples, while the direction and magnitude of shared DEG responses remained highly consistent between the two analyses. Thus, reference-genome choice primarily influenced the representation and detection of a subset of individual genes rather than the overall transcriptional response. Together, these findings provide a refined framework for understanding temporal transcriptional and biochemical adjustment during cold treatment and recovery in winter rapeseed and offer candidate genes and processes for future functional studies.

## Data Availability

The data presented in this study have been deposited in the NCBI Sequence Data Bank under accession number SRP179662.
