# Supplementary material for: Temporal and genotype-dependent transcriptomic responses to cold treatment and recovery in winter rapeseed (Brassica rapa L.)
Source: Front Plant Sci. 2026 Sep 17;17:1947775. doi: 10.3389/fpls.2026.1947775 (PMC13627060; doi:10.3389/fpls.2026.1947775)
Supplement: Supplementary file 1 [file SupplementaryFile1.docx]

Supplementary Material

## 1.1 Supplementary Figure

**Supplementary Figure 1.** GO and KEGG enrichment analyses of DEGs between L7 and Lenox. (A) GO enrichment analysis of DEGs in the CK comparison. (B) GO enrichment analysis of DEGs in the Re24h comparison. (C) KEGG enrichment analysis of DEGs in the CK comparison. (D) KEGG enrichment analysis of DEGs in the Re24h comparison.

**Supplementary Figure 2.** KEGG enrichment analyses of genes upregulated within Longyou-7 and Lenox relative to their respective genotype-matched controls. (A–C) Top-ranked KEGG pathways associated with genes upregulated in Longyou-7 at 3 h (A), 24 h (B), and Re24h (C) relative to the Longyou-7 CK. (D–F) Corresponding pathways associated with genes upregulated in Lenox at 3 h (D), 24 h (E), and Re24h (F) relative to the Lenox CK. Bubble size represents the number of upregulated DEGs assigned to each pathway, bubble color represents the nominal enrichment P-value, and the horizontal axis shows the enrichment score. Up to 20 pathways ranked by nominal P-value are displayed for each contrast.

**Supplementary Figure 3.** KEGG enrichment analyses of genes upregulated during recovery within Longyou-7 and Lenox. DEGs were identified by comparing Re24h with 24 h separately within each cultivar. (A) Top-ranked KEGG pathways associated with genes upregulated during recovery in Longyou-7. (B) Corresponding results for Lenox. Bubble size represents the number of upregulated DEGs assigned with each pathway, bubble color represents the nominal enrichment P-value, and the horizontal axis shows the enrichment score. Up to 20 pathways ranked by nominal P-value are displayed for each cultivar.

**Supplementary Figure 4.** Expression-pattern clustering of DEGs and functional enrichment analysis of representative clusters.

**Supplementary Figure 5.** Functional enrichment analyses and expression heatmaps of representative expression-pattern clusters. (A) GO enrichment analysis of cluster a. (B) GO enrichment analysis of cluster e. (C) KEGG enrichment analysis of cluster a. (D) KEGG enrichment analysis of cluster e. (E) Heatmap of standardized expression levels of genes in cluster a across treatment conditions. (F) Heatmap of standardized expression levels of genes in cluster e across treatment conditions.

**Supplementary Figure 6.** RT-qPCR assessment of the expression patterns of 14 additional candidate genes. (A–N) Expression profiles of the 14 candidate genes across the eight cultivar-by-stage groups. Green dashed lines indicate relative expression measured by qRT-PCR, and orange dashed lines indicate RNA-seq expression based on FPKM values. Insets show the qRT-PCR relative expression levels across the eight groups; values are presented as the mean ± SD of three independent biological replicates. Different lowercase letters indicate significant differences among groups based on one-way ANOVA followed by Duncan’s multiple range test (*P* < 0.05); groups sharing at least one letter are not significantly different.

**Supplementary Figure 7.** Conserved motif and domain architectures of GST-related candidate proteins identified under the Longyou-7 and Chiifu v1.5 reference backgrounds.(A) Phylogenetic relationships, conserved motifs, and GST domain architectures of 17 GST-related candidate proteins identified under the Longyou-7 reference background. The conserved motifs are indicated by colored boxes, and the GST_N and GST_C domains are shown in green and yellow, respectively. Conserved-domain analysis supported classification of the 17 candidates into nine Tau and eight Phi GSTs. (B) Phylogenetic relationships, conserved motifs, and GST domain architectures of 26 GST-family candidates retained from the Chiifu v1.5 reference background after conserved-domain screening. Colored boxes indicate the conserved motifs identified among the analyzed proteins, whereas GST_N and GST_C domains are shown in green and yellow, respectively. Conserved-domain analysis supported predominantly Phi-class assignments among the Chiifu candidates, together with several Zeta-class proteins. Protein lengths and motif/domain positions are shown according to amino-acid coordinates.

**Supplementary Figure 8.** Comparative phylogenetic relationships of GST-related protein sequences from four plant materials representing three species. The phylogenetic tree was constructed using 126 protein sequences, including 19 sequences from Arabidopsis thaliana (AT), 32 sequences from Chinese cabbage (Brassica rapa subsp. pekinensis ‘Chiifu’; Bra), 17 candidate sequences from winter turnip rape (B. rapa ‘Longyou-7’; Brapa), and 58 sequences from Brassica napus (Bna). Sequence names are indicated by the corresponding prefixes around the tree. Colored outer ranges indicate the GST subclasses/groups used in the phylogenetic visualization, including GATZ+GSTT, Phi, Lambda, DHAR, and Tau.

**Supplementary Figure 9.** CDS sequence alignments of Longyou-7 candidate genes with their best-matching sequences in the Chiifu v1.5 gene set. TBtools was used to identify the sequences showing the highest similarity to the Longyou-7 candidate genes in the Chiifu v1.5 CDS dataset and to perform nucleotide sequence alignments. (A) Sequence alignment between *Brapa10G002988* and *Bra032578*, with 74.56% identity. (B) Sequence alignment between *Brapa09G004869* and *Bra027840*, with 83.51% identity.

**Supplementary Figure 10.** Hub-gene co-expression subnetworks of the Longyou-7 coral1 and darkslateblue modules.(A) Simplified co-expression subnetwork of the top 10 hub genes in the coral1 module. (B) Simplified co-expression subnetwork of the top 10 hub genes in the darkslateblue module. Hub genes were ranked using the MCC algorithm in cytoHubba and visualized in Cytoscape. Node colors indicate relative hub ranking, with warmer colors representing higher-ranked hub genes. Edges represent co-expression connections among the selected genes.

## 1.2 Supplementary Tables

**Supplementary Table 1.** Quantitative overlap and effect-size concordance of differential-expression calls obtained using the Longyou-7 and Chiifu v1.5 reference genomes across six within-genotype contrasts.

|  | **DEG calls (gene models)** | | **Best-hit DEG-group overlap** | | | | **Shared best-hit pairs** | | |  |
| --- | --- | --- | --- | --- | --- | --- | --- | --- | --- | --- |
| **Contrast** | **L7 ref.** | **Chiifu ref.** | **Shared** | **L7 only** | **Chiifu only** | **Jaccard** | **n** | **Direction concordance**  **(%)** | **Pearson r** | **L7 no-hit DEGs, n**  **(%)** |
| L7 3 h vs L7 CK | 1,465 | 732 | 611 | 836 | 121 | 0.390 | 622 | 99.84 | 0.991 | 49 (3.34) |
| L7 24 h vs L7 CK | 6,922 | 5,357 | 4,555 | 2,184 | 802 | 0.604 | 4,692 | 99.62 | 0.986 | 173 (2.50) |
| L7 Re24h vs L7 CK | 1,684 | 1,085 | 912 | 745 | 173 | 0.498 | 926 | 99.78 | 0.967 | 52 (3.09) |
| Lenox 3 h vs Lenox CK | 2,832 | 1,730 | 1,489 | 1,292 | 241 | 0.493 | 1,519 | 99.93 | 0.992 | 61 (2.15) |
| Lenox 24 h vs Lenox CK | 5,646 | 3,955 | 3,429 | 2,075 | 526 | 0.569 | 3,530 | 99.41 | 0.984 | 124 (2.20) |
| Lenox Re24h vs Lenox CK | 2,231 | 1,347 | 1,148 | 1,038 | 199 | 0.481 | 1,178 | 99.66 | 0.994 | 46 (2.06) |

**Note.** DEGs were re-filtered using harmonized criteria of adjusted P < 0.05 and |log₂FC| ≥ 1. Longyou-7 gene models were linked to retained Chiifu v1.5 targets using one-direction BLASTn best-hit mapping. The Jaccard index was calculated as Shared/(Shared + Longyou-7 only + Chiifu only). Direction concordance and Pearson’s r were calculated for Longyou-7 DEG genes whose retained Chiifu v1.5 best hit was also identified as a DEG. “L7 no-hit DEGs” denotes Longyou-7-reference DEG models without a retained Chiifu v1.5 BLAST hit under the applied comparison settings. L7, Longyou-7; Re24h, 24 h after return to the control temperature following 24 h of cold treatment.

**Supplementary Table 2.** Functional context of Longyou-7-reference DEGs without a retained Chiifu v1.5 BLAST hit across six within-genotype contrasts.

| **Contrast** | **No-hit DEGs**  **(n)** | **Percentage of L7-ref DEGs** | **FDR-significant KEGG terms among no-hit DEGs** |
| --- | --- | --- | --- |
| L7 3 h vs L7 CK | 49 | 3.34% | Not evaluated from the verified 49-gene set |
| L7 24 h vs L7 CK | 173 | 2.50% | Carotenoid biosynthesis (ko00906; q = 0.0133; 2 genes) |
| L7 Re24h vs L7 CK | 52 | 3.09% | None |
| Lenox 3 h vs Lenox CK | 61 | 2.15% | None |
| Lenox 24 h vs Lenox CK | 124 | 2.20% | Carotenoid biosynthesis (ko00906; q = 0.0376; 1 gene); DNA replication (ko03030; q = 0.0376; 1 gene) |
| Lenox Re24h vs Lenox CK | 46 | 2.06% | None |

**Note.** Only KEGG terms with q < 0.05 are listed. The 3-h Longyou-7 KEGG result is not reported because the available enrichment output was not generated from the verified 49-gene intersection. The absence of a retained BLAST hit reflects the supplied sequence-comparison settings and may arise from sequence divergence, gene-model differences, or annotation completeness; it does not by itself establish gene novelty or genomic absence.
