# Supplementary material for: Temporal and genotype-dependent transcriptomic responses to cold treatment and recovery in winter rapeseed (Brassica rapa L.)
Source: Front Plant Sci. 2026 Sep 17;17:1947775. doi: 10.3389/fpls.2026.1947775 (PMC13627060; doi:10.3389/fpls.2026.1947775)
Supplement: Supplementary file 2 [file SupplementaryFile2.docx]

>Bra037976.1

MAYASRILNHSKKLKDVSTLLRREQAASIRYFSSTNRAPLSREDAFSAHLGFSPVERMTK

CSTDIVSICFSNTRTTLRSRVGRPRLGNHFSCSMQSVRGFSSGSDLPPHQEIGMPSLSPT

MTEAGNIAKWLKKEGDKVAPGEVLCEVETDKATVEMECMEEGFLAKIVKAEGSKEIQVGE

VIAITVEDEEDIGKFKDYTPSSTADASPPKADAAPPKTDAAPPPPKEEKASPPEPKTSKP

GPPPTGDRVFASPLARKLAEDNNVSLSSIKGTGPEGRIVKADIEDYLASGGKEATAKQSK

VTDSKVPALDYVDIPHSQIRKVTASRLAFSKQTIPHYYLTVDACVDKLMGLRSQLNSFQE

ASGGKRISVNDLVVKAAALALRKVPQCNSSWTDDYIRQFKNVNINVAVQTENGLYVPVVK

DADKKGLSTIGEEVRLLAQKAKENTLKPEDYEGGTFTVSNLGGPFGIKQFCAVVNPPQAA

ILAVGSAEKRVVPGNGPDEYNFASYMPVTLSCDHRVVDGAIGAEWLKAFKGYIENPESML

L

>Bra037204.1

MEDQKMKLHCGFIFSNWTAMFGLHEKSLDFELVFVDWVAGEARTKTFLSNLNPFGQVPVL

EDGDLKLFESKAITRYLAEQYKDVGTDLLPNDAKERAIVSMWMEIDTNQFLPLASTLIRE

LVTKPYQGLATDFTSVQENKEKLSEVLNIYEARLGESCYLAGESFTLADLHHLPPIHYML

KMEEEGVKDLIYSRPNVTAWVEKMQSRPAWLKTVVKGDHIFELMKQRRMPMQLDSPCQEV

TGLAQKTAMVTENT

>Bra036260.1

MASIKVFGHATTTSTRRVLLTLHEKNLDYELVNVELKDGEHKKEPFLSRNPFGQVPAFED

GDLKLFESRAITQYIAHRYEDQGTNLLPADSKNIAQYATMSIGMEVEAHQFDPAASKLAW

EQLFKHIYGLTTDQAVVAEQEAKLAKVLDVYEARLKEFKYLAGETFTLTDLHHIPVIQYL

LGTPTKKLFTERPRVNEWVAEITKRPASQKILQ

>Bra036259.1

MAGIKVFGNATSPSTRRVLLALHEKNLDFELVNIDLKDGEHKKEPFLSRNPFGKVPAFED

GDLKLFESRAITQYIAHRYESQGTNLLPADSKNPAHYAIMAIGMEVEAHQFDPVASKLVW

EQVIKNFVGLTTDQAVVAEEEAKLVKVLDVYEARLKEFKYLAGETFTLTDLHHIPVVQYL

LGTPTKKLFTERPRVNEWVAEVTKRPASQKILK

>Bra033361.1

MSNTSHPFMDCLKMVFNLFPNWKRATEVKKLESRAITQYIAYVHSSRGTQLLNLQSHETM

AILTMWMEIEAHQFDPLASKLTWELVIKPLYGLETDHMVVKENEAGLEKVLDVYEKRLGE

SRFLACNTFTLVDLHHLPNIQFLLGTPTKRLFENRPKVRNWVHEITSREAWKMACDPEKS

WFVNQGK

>Bra033360.1

MSSSKLSVTLALMIVIMSISYEAYALAHKDGYKIYGYPSSPNTRRALAVLHEKGLSFDRI

TVNLTTGDPQKPSFLPINPFGQVPVFLDGHLKLIEQTIKPLKGLKADYKVVNETETNLGK

VLDVYEERLKNSRFLASNRFTLADLFHLPNIEYLMNTTTKRLFETRPNVHRWVAKITARP

AWKKACDAKAWYDKKKN

>Bra033359.1

MRFSRLLVTFSLMMMMAISNEPFALAHKEEYKIYGFPYSASTWRVLAVLHEKGLSYYPIT

VNLRTGEQKKPSFLSINPFGQVPVLLDGHLKLTESRAISLYIESAHRSRGTKLLNHKNYK

KMGIETMWMYIESFEFDPPATTLTFEQAIKPMTGLKTDYKVVNETEPQLEKVLDIYEERL

KNSRFLAGNRFTLADLFHLPNIEYLMNTTTKRLFESRPNVHRWVAKIMARPAWRKACDAN

AWYDEMEN

>Bra033358.1

MAGIKVFGHPASTATRRVLIALHEKELDFELVHIDLKDGEHKKEPFLSRNPFGKIPAFED

GGFKLFESRAITQYIAHEYADKGNQLLSPGSKNMAILAMGMEIEAHEFDSVASKLGWEQI

FKNFFGLTTDQAVVKEEEVKLGKVLDNYEARLGESKYLACDHFTLVDLHHIPVIQYLLGT

PTKKLFDERPHVSAWVADITSRPSSQKVLL

>Bra032578.1

MNWTKKVYRLVPNWKRETEVKNLGYKVHGNPFSTSTRRVLAVLLEKGLSYESITVDLKSG

EHKTESFLSLNPFGQIPVFVDGNIKLHESRAITQYIAYVHSTRGTQLLYLQSHETMAILT

VWMEIEAHQFDPFASKLTWEQAIKPIYGLETDQVVVRENEANLEKVLDIYEKRLGKYRFL

VCNTFTLADLHHLPNIQYLLGTPTKRLFEDRPKVLKWVGEITGREAWKMACDHEKYWFGK

QTK

>Bra032010.1

MVVKLFGQIKAGNPQRVLLCFLEKGIEFEVIHVDLNTFEQKKPEYLLRQPFGQVPAIEDG

DLKLFESRAISRYYATKYADQGTDLLGKTLEQRAIVEQWMEVEANYFNVVVLPLVINIVF

TPKFDVALVEELKVKLDKVLDVYENQLAMNRYLAGAEFSLADLTHMPGMRYIMNEAGLGS

MITSRENVNRWWNEMSARPAWKKLMEMAAY

>Bra030515.1

MAGIKVFGHPASTATRRVLIALHEKDLDFELVHVELKDGEHKKEPFLSRNPFGKVPAFED

GDFKLFESRAITQYIAHEYADKGNQLLSPGSKNMAILAMGMEIEAHEFDSVASKLGWEQI

FKNFFGLTTDQAVVKEEEVKLGKVLDNYEARLGESKYLASDHFTLVDLHHIPVIQYLLGT

PTKKLFEERPHVSAWVADITSRPSSQKILL

>Bra028057.1

MSRLLNTSFLPSSTCRTRSCSSSAPHFRGNKPRVVTVRAKIREIFMPALSSTMTEGKIVS

WVKSEGDKLSKGESVVVVESDKADMDVETFYDGYLAAIMVEEGGVAPVGSAIALLAETEE

EIADARAKASLGGGGTSPVSQAPPVVEDTVSSVPPLKAVSSASASASAVHPASEGGKRVV

ASPYAKKLAKELKVELAALVGSGPMGRIVAKDVEAAVAAAPPPPVQKEPSVELGSVVPFT

TMQAAVSRNMAESLSVPTFRVGYTINTDALDALYKKIKSKGVTMTALLAKATALALAKHP

VVNSSCRDGNSFVYNSSINIAVAVAIDGGLITPVLQNADKVDIYSLSRKWKELVDKARAK

QLQPQEYNTGTFTLSNLGMFGVDRFDAILPPGTGAIMAVGASQPTVVATKDGRIGMKNQM

QVNVTADHRVIYGADLAQFLQTLASIIEDPKDLTF

>Bra026601.1

MAYTSEEMKEKLKLYSYWRSSCGHRVRIALSLKGIEYEYIPVNLLKGEQFDPDFMKINPM

GTVPALVDGEVVITDSFAIIMYLDEKYPEPPLLPRDLHKRALNYQVASIISSGIQPHQNL

AVIRYIEEKTNGEEKIAWINNAIRKGYTALEKLLVNYAGIYATGDEIYLADLFLVAQVHG

AMIKFKIDVQNIWTTKLNSPNLVLPKPIFFEGMIRPLLVFDGWVSLSDISGGYTENSSCC

IMPYLSCGMILLVGSPGLVSISPSFSSEKCPLSPSLLSVKGDVFLVSLPSISFSFFTGLL

SCGAVCTGPEDAIKITHVFLVGESWLSTSLVTISQLSDFVVKALSTQSSFVLISLSSSLE

ELSILTYFEPYPTLAKCYESYKELSVFEDAVPAKQPDAPAPMI

>Bra025167.1

MALSSSSFLSTPSLPNSKSTISFASSVSPAPIPSLRRVVFRSHRREMTVRSKIREIFMPA

LSSTMTEGKIVSWLKTEGEKLAKGQSVVVVESDKADMDVETFYDGYLAAIVVGEGETAPV

GAAIGLLAETEAEIEEAKSKAASKSSSAAVAPSPPPPATSSPAPAISQPAPVAAGSDGPR

KTVATPYAKKLAKQHKVDIGSVAGTGPFGRITASDVEAAAGIAPSVAPPPPPPPAAPTAT

TAKATTTSSPPLLPDSSVVPFTAMQSAVSKNMIESLSVPTFRVGYPVNTDALDALYEKVK

PKGVTMTALLAKAAGMALAQHPVVNASCKDGKSFTYNSNINVAVAVAINGGLITPVLQDA

DKLDLYLLSQKWKELVGKARSKQLQPHEYNSGTFTLSNLGMFGVDRFDAILPPGQGAIMA

VGASKPTVVADKDGFFSVKNKMMVNVTADHRIVYGADLAAFLQTFAKIVENPDSLTL

>Bra024820.1

MKLYSYWRSSCAHRVRIALSLKGLEYEYIPVNLLKGEQSDPDFKKINPMGTVPALVDGDV

VISDSLAIIMYLDEKYPEPPLLPPDLHKRAVNFQAASIVLSGIQPHQNLGVIKFIEEKIN

SEEKTAWVTNAITKGFTALEKLLVSCAGKHATGDEVYLADLFLAPQIYGAINRFQINMEP

YPTLAKCYESYKDLPAFQNAAPEKQPDAPASTS

>Bra024517.1

MLLLMQPFGQVPVFEDENVKLFESRAITQYIAYLHSARGTQLLSLESEETLATLTMWMEI

EAHQFDPFASRLTWEQVIKPLYGLETDHTVVKENEAGLEQVLNVYEKRLGESRFLACNTF

TLVDLHHLPNIQYLLETPTKGLLENRPRVRMWVDEITSKNRRHLLSFHQFLQDIIQNL

>Bra023602.1

MVVKLYGLVTAACPQRVLLCFLEKEIEFEIVHVDLDTLEQKKPEHLLRQPFGQVPAIEDG

DFKLFESRAIARYYATKYSNQGTNLLGKSLEHRAIVDQWADVETHYFNVLAFPIVLNLVI

KPRLGEECDAVLVEELKAKLGVVLDIYENRLASNRFLAGDEFTMADLTHMPAMGYLMRTD

VNQMVKARVNMNRWWEELTARPAWKKLMKMAGFEA

>Bra022816.1

MVLTIYAPLFASSKRAVVTLVEKGVEFETVNVDLMKGEQRQPEYMAIQPFGKIPVLVDGD

YKIFESRAIMRYIAEKYRSQGPDLLGKTIEERGQVEQWLDVEATSYHPPLLALTLNIVFA

PLMGFPADEKVIKESEEKLGEVLDVYEAQLSKNEYLAGDFVSLADLAHLPFTEYLVGAIG

KAYMIKDRKHVSAWWDKISNRAAWKEVSEKYALPV

>Bra022815.1

MVLKVYGPHFASPKRALVTLIEKGVAFETVPVDLMKGEHKQPAYLALQPFGTVPAVVDGD

YKIFESRAVMRYVAEKYRSQGPDLLGKTVEERGQVEQWLDVEATTYHPPLLNLTLHIMFA

SVMGFPSDEKLIKESEEKLSAVLDVYEAHLSKSKYLAGDFVSLADLAHLPFTDYLVGPIG

KAYMIKDRKHVSAWWDSISSRPAWKETLEKYSFPA

>Bra021673.1

MVLKVYGPHFASPKRALVTLIEKGVPFETVPVDLMKGEHKQPAYLALQPFGTVPAVVDGD

YKIFESRAVMRYVAEKYRSQGPDLLGITVEDRGQVEQWLDVEATTFHPPLLNLTLHIMFA

SVMGFPSDEKLIKESEEKLSAVLDVYEAHLAKSKYLAGDFVSLADLAHLPFTDYLVGPIG

KAYMIKDRKHVSAWWDDISSRPAWKETLEKYSFPA

>Bra018543.1

MAGIKVFGHAASTATRRVLLTLHEKNLDFELVHVELKDGEHKKEPFLSRNPFGKVPAFED

GDLKLFESRAITQYIAHRYEEEGTNLLPADSKNISHYAIMAIGMEVEAHHFDPVASKLAW

EQVFKLFYGMTTDQAVVEEEEAKLATVLDVYEARLKEFKYLAGETFTLTDLHHIPVIQYL

LGTPTKKLFDERPHVSEWVAEITNRPASQKILQ

>Bra017417.1

MVISDHRLYSISSHSKYIFPCDQAEKKEKLKLYSYWKSSCSHRVRIALTLKGIEYEYVPV

NPLKRDEFLKINPMGAVPALVDGDVVVSDSLAIIMYLDEKYPEPPLLPRDLHKRAVNYQA

ASIVFSGIQPYQNTPVARYIEEKTNAEEKTAWVSNAITKGFTALEKLLVSCTGKYATGDE

VYLADLFLAPQIHAAINRFQINMEPYPTLAKCYESYNDLPAFQNAVPEKQPDAPASTS

>Bra017345.1

MAASSSFLSTASLSNPKSTISFASSVSPAPIPSLRRVVFRSPSSHRRVMTVRSKIREIFM

PALSSTMTEGKIVSWIKTEGEKLAKGESVVVVESDKADMDVETFYDGYLAAIVVGEGETA

PVGAAIGLLAETEAEIEEAKNKAASKPSSSAVVPSPPPATSSPAPAIAQPSPAAAASDGP

RKTVATPHAKKLAKQHKVDIGSVAGTGPFGRITASDVEAAAGIAPTVTPPPPPPPPAAAP

TPTAKATTTSSPPLLPDASIVPFTAMQSAVSKNMIESLSVPTFRVGYPVNTDALDALYEK

VKPKGVTMTALLAKAAGMALAQHPVVNASCKDGKSFSYNSNINVAVAVAINGGLITPVLQ

DADKLDLYLLSQKWKELVGKARNKQLQPHEYNSGTFTLSNLGMFGVDRFDAILPPGQGAI

MAVGASKPTVVADKDGFFSVKNKMLVNVTADHRIVYGADLAAFLQTFAKIVENPDSLTL

>Bra015679.1

MTGSFDVAVRSHCSGMWRGGDTDKNLTRDRDDSEFMRRTSRQLIANASVDDEILVLTSKG

LNTPLLDNSFGLGSEAMQLYHHPYSLDSQRVRLALEEKGIDYTSYHVNPITGKHMDSSFF

RMNPNAKLPVFRNGSHIILDTIEIIEYLERIAEVSSGVEDATFGREVLEWMRKIREWDSK

LFTLAHIPDNRRLYVSKFLRMVMIARMAESPDLASAYHRKLREAYDTEDKLKDPEALRRS

KDHLLRLLDEVETKLEGTSYVAGNEFSMADVMLVPVLARLSLLDLEEEYISSRRNLAEYW

VVVRSRPSYKKVVGRYFNGWRKYATLLKTWMFVRVRSLLRKY

>Bra014394.1

MAMKVYGNGMSVCVARVLLCLHEKETEFELVPVDLFACHHKLPSFLSMNPFGQVPVLQDD

DLTLFESRAITAYIAEKHKDKGTDLTRHADAKEAAIVKLWSEVESHHFNPAISAVIHQLI

VVPLQGKTPDAAIVEENLEKLGKVLDVYEEKLGKTKYLAGDSYTLADLHHVPYTYYFMKT

CHAGLVNDRPKVKAWWEDLCSRPAFLKVSPGLTVAPPTN

>Bra008570.1

MVVKLYGQVTAACPQRVLLCLLEKEIEFEIIHIDLDTLEQKRPEHLLRQPFGQVPAIEDG

DFKLFESRAIARYYATKYADQGTNLLGKSLEHRAIVDQWADVEVGYFNVLVHPLVMNLVI

KPRLGEECDAVLVEELKVKLGVVLDIYENQLASNRFLAGDEFTMADLTHMPAMGYLMRTN

INRIVKDRVNMNRWWEEVTARPAWKKLMKMAGF

>Bra006486.1

MLNGLCDFLKICRGRVRRLRWEPRSVCWRRLRLRSIEEPQSKAASKSSSASPVVVPSPPP

PTASSPAPAIAQHAPVATVSDGPRKTVATPYAKKLAKQHKVDIRSVAGTGPFGRITASDV

EAAAGIAPSVAAPPPPVVNASCKAQRWSTTATGHQQGNNTLVTNKMYSLQPIHVMAGQEI

SGKLHLVVHRKSVGPSADKGGILHTSSCKFNLKEPYYRMSQPSCTRTTSTTVKVKLASTL

DHIGNVAQLSPLIGFVHKHVVIVDTEPELEKSEFLVMSLEKCVKQLEEERATEETYHHQQ

RKCKL

>Bra006098.1

MLNVNFSTQVGKQRGSVDIRSVAGTGPFGRITASDVEAAAGIAPSVAAPPPPPPPAAAAK

ATTTSSSSPPLLPGSSVVPFTAMQSDVSKNMIESLFVPMFRVGYPVNTDALDAPYEKVKP

KGVLLSTINWAICKAIDLLELLKIEEEMLSAQSRKTVKRSTSWCNDNSLRREEEDKEDNA

EDEEAVMKARAVDDWKNYNPCGACNQKLTTCD

>Bra004434.1

MRVLATLYEKGLDFELVPVDMKAGAHKQEPLISLNPFGQIPALLDGDLTLFESRAITEYI

ADEYNNKGEKLLCQSCKKLKAITKVWLQVEGQQFDPIASKLAFERVFKGMLGMTTDPATV

EDLEAKLVKVLDIYEARLSKSPILAGDCFTLADLHHLPIIYYLMGTDSKKLFESRPKVSE

WIKKITARPSWVKVLNLQKQ

>Bra000876.1

MAGIKVFGHAASISTRRVLLTLHEKNLDFELVHVDLKDGEHKTESFLSHNPFGKVPAFED

GDLKLFESRAITQYIAPRYEGQGTNLLQPDSKNLAQYAIMAMGMQVEAHQFDPLASKLAW

EHVFKLIYGLTTDQAVVAEEEAKLAKVLDVYEARLKEFKYLAGDTFTLTDLHHIPAVQYL

LETPTKKLFTERPHVNKWVAEITKRPASQKILQ

>Bra000875.1

MAGIKVFGHPASAPTMRVLLTLHEKNLDFEFVHVDLMGGEHKKEAFLARNPFGQVPAFED

GDLKLFESRAITQYIAHRYEGQGTNLLPADSKNIAHYAIMAIGMQVEAHQFEPVAAKLVS

EQVFKLKKGLTTDQAVVAEEEAKLDKVLDVYEARLKEFKYLAGDTFTLTDLHHIPTVKYL

LGTPTKKLFTERPRVNEWVAEITKRPASQKILQ

>Bra000460.1

MASIKVHGVPMSTATMRVLAALYEKELEFELIPVDMRAGAHKQEPFLSLNPFGQIPALQD

GDLTLFESRAITEYIGDEYSEKGEKLMCPGCNKVKALTKVWLNVEGQQFDPIASKIAFER

IFKGMFGMTTDPAAVQELEGKLVRVLDIYEARLSKSEFLACDCFTLADLHHLPVIHYLMG

TDSKALFESRPKVCEWVKKITARPAWAKVVDLQKQ
