# Supplementary material for: Temporal and genotype-dependent transcriptomic responses to cold treatment and recovery in winter rapeseed (Brassica rapa L.)
Source: Front Plant Sci. 2026 Sep 17;17:1947775. doi: 10.3389/fpls.2026.1947775 (PMC13627060; doi:10.3389/fpls.2026.1947775)
Supplement: Supplementary file 3 [file SupplementaryFile3.docx]

>Brapa02G000698.1

MVVKLYGQVTAACPQRVLLCFLEKEIEFEIVHVDLDTLEQKKPEHLLRQPFGQVPAIEDG

DFKLFESRAIARYYATKYSNQGTNLLGKSLEHRAIVDQWADVETHYFNVLAFPIVLNLVI

KPRLGEECDAVLVEELKAKLGVVLDIYENRLASNRFLAGDEFTMADLTHMPAMGYLMRTD

VNQMVKARVNMNRWWEELTARPAWKKLMKMAGFEA

>Brapa02G002033.1

MGLISDEYVRLLGAWPSPFVLRTRIALNLKRVPYEYLEEEDSLNSESVLNYNPVHKQIPI

LIHGNKPIRESLNIVMYVDETWLSGPPILPSDPFDRAVARFWDVYIDEHCFTSINGVAVA

KDEEERKAAIAKLEMCMALLEETFQECSKGRGFFGGDNIGFIDIGFGSMLGPLKVLEKFT

GVKFIHPETTPGLFHWVDRFYSHEAVKPVMPDIEKLVEFARLKFNTSIFK

>Brapa03G002459.1

MMRTILQSQLLPPPPIKLRHSSLLFSPSQHSRPNLKFTSKSSPSPPTITMASIKVHGVPM

STATMRVLAALYEKELEFELIPVDMRAGAHKQEPFLSLNPFGQIPALQDGDLTLFESRAI

TEYIGDEYSEKGEKLMCPGCNKVKALTKVWLNVEGQQFDPIASKIAFERIFKGMFGMTTD

PAAVQELEGKLVRVLDIYEARLSKSEFLACDCFTLADLHHLPVIHYLMGTDSKALFESRP

KVCEWVKKITARPAWAKVVDLQKQ

>Brapa03G002934.1

MAGIKVFGHPASAPTMRVLLTLHEKNLDFEFVHVDLMGGEHKKEAFLARNPFGQVPAFED

GDLKLFESRAITQYIAHRYEGQGTNLLPADSKNIAHYAIMAIGMQVEAHQFEPVAAKLVS

EQVFKLKKGLTTDQAVVAEEEAKLDKVLDVYEARLKEFKYLAGDTFTLTDLHHIPTVKYL

LGTPTKKLFTERPRVNEWVAEITKRPASQKILQ

>Brapa03G002935.1

MAGIKVFGHAASISTRRVLLTLHEKNLDFELVHVDLKDGEHKTESFLSHNPFGKVPAFED

GDLKLFESRAITQYIAHRYEGQGTNLLQPDSKNLAQYAIMAMGMQVEAHQFDPLASKLAW

EHVFKLIYGLTTDQAVVAEEEAKLAKVLDVYEARLKEFKYLAGDTFTLTDLHHIPAVQYL

LETPTKKLFTERPHVNKWVAEITKRPASQKILQ

>Brapa04G002288.1

MAERSEEVKLLGMWASPFSRRVEIALTLKGVPYEFSEQDITNKSPLLLQSNPVHKMIPVL

LHNGKPISESLVILEYVDDTWQNNPILPQDPYDRATARFWARFVDEQVYVTAMKVVGKVG

EEKDAAVEATRDLLKLLEKELVGKDFLGGESLGFVDIVATLVAFWLMRTEEVVGVKVVPV

EMFPEIHRWVKNLLDVDVIKKCIPPEDEHLDYIRARMESLKLKSV

>Brapa05G001498.1

MAEKEEVKLLGIWASPFSRRIEMALKLKGVPYEYVEEILEKKSPLLLALNPIHKKIPVLV

HNGKTILESQVILEYIDETWKHNPLLPRDPYERSKARFLAKLVDEQIITAGFVSMARADE

KGREVLAEQTRELIMNLEKELVGKDFFGDKTVGFLDLVAGSVIPFCLERGWEGIGLEVIS

EDKFPEYKRWVKNLEKVDFVKDCIPPKEKHVEHMNHMGERIRSA

>Brapa05G003915.1

MVVKLFGQIKAGNPQRVLLCFLEKGIEFEVIHVDLNTFEQKKPEYLLRQPFGQVPAIEDG

DLKLFESRAISRYYATKYADQGTDLLGKTLEQRAIVEQWMEVEANYFNVVVLPLVINIVF

TPKFDVALVEELKVKLDKVLDVYENQLAMNRYLAGAEFSLADLTHMPGMRYIMNEAGLGS

MITSRENVNRWWNEMSARPAWKKLMEMAAY

>Brapa07G001551.1

MAQNDTVKLIGSWASPFSIRARAALHLKSVKYQYSDEPDVLRSKSELLLKSNPIFKKVPV

LIHGDVSICESLNIVQYIDEAWSSGPSILPSHPVERANARFWALFIDEKIFGSLEAVGGA

KDDEGRMAAAGKLMENLAILEEAFQKSSKGLGFFGGENIGFLDLACGTLLGPVSVIEAFS

GVKFLRQETTPGLIQWAEKFRAHEAVKPYMPTPEEFVAFAKKKFNVE

>Brapa07G002845.1

MADEVILLDFWPSPFGVRARIALREKGVEFEYREENLREKSPLLLQMNPVHKKIPVLIHN

GKPVCESMNVVQYIDEVWSGKNPLLPSDPYQRAQARFWVDFIDTKVYEPADKIWSTKGEV

QEKAKKEYIEALKILESELGNKPYFGGDNFGFVDIAMTGYYMWFEASEKCGNFSIEPECP

ILMASAKRCLQRDSVVNSVPDPEKIVEFAFKIRKIYCV

>Brapa07G003700.1

MGLLSGVNNDEYVRLLGAWPSPFVLRTRIALNLKRVPYEYLEEEDTLNSESVLNYNPVHK

QIPILIHGNKPIRESLNIVMYVDETWLSGPPILPSDPFDRAVARFWDVYIDEHCFTSING

VAVAKDEEERKMAIAKLEQCMALLEETFQECSKGRGFFGGDNIGFIDIGFGSMLGPLKVL

EKFSGVKFIHPENTPGLFNWADRFYAHEAVKPVMPDIEKLVEFARLKFNTSIFK

>Brapa08G002991.1

MADEVILLDYWPSMFGMRTKMALAEKGVSYEYIETDPWIKTPLLIEMNPIHKKIPVLIHK

GKPICESLIQLEYIDEVWSGTYPMLPSDPYQKAQARFWADFIDKKFYDPSWKVWGTNGEE

QVAAKKELLEHFKTLETELGDKTYYGGEVFGFLDIALMGYYSWFKAMEKFGEFSIETEFP

KLTEWTKRCLERESVVKALTDSDKILEYAYVLRKKFGAE

>Brapa09G000849.1

MADEVILLDYWPSMFGMRTRVALEEKNIKFDYREQDLFNKSPILLEMNPVHKKIPVLIHN

GKPVCESLIQVEYIDETWPGGNPFLPSDPYQRAQAKFWGDFIDKKVYGPTRLIWGAKGEE

QEAGTKEFIEMLKMLESELGDKTYFGGETFGYVDIAMIGFYCWFDVLEKCGNFSIEAECP

KLIAWAKRCMKRESVAKSLPDSNKITKFVPELKKKIGIE

>Brapa09G004869.1

MGERQEVKLLGTWYSPVVARAKIALRLKSVDYDYFEEDLFGSKSELLLKSNPVHKKVPVL

IHNNKPILESLNIVEYIDETWNASGPSILPSHPRDRSQARFWSAFVDDKWFPTLIATTIT

KSKNAKAKGMEEVGEGLLLLEDAFISISKGKRFFGGEVIGFMDICLGSFLVILKGREKLN

GENILDKSKTPSLCKWANEFLADDTVKNVLPEIDRVVKFMREFN

>Brapa09G005606.1

MEDQKMKLHCGFIFSNWTAMFGLHEKSLDFELVFVDWVAGEARTKTFLSNLNPFGQVPVL

EDGDLKLFESKAITRYLAEQYKDVGTDLLPNDAKERAIVSMWMEIDTNQFLPLASTLIRE

LVTKPYQGLATDFTSVQENKEKLSEVLNIYEARLGESCYLAGESFTLADLHHLPPIHYML

KMEEEGVKDLIYSRPNVTAWVEKMQSRPAWLKTVVKGDHIFELMKQRRMPMQLDSPCQEV

TGLAQKTAMVTENT

>Brapa09G006420.1

MAGIKVFGNATSPSTRRVLLALHEKNLDFELVNIDLKDGEHKKEPFLSRNPFGKVPAFED

GDLKLFESRAITQYIAHRYESQGTNLLPADSKNPAHYAIMAIGMEVEAHQFDPVASKLVW

EQVIKNFVGLTTDQAIVAEEEAKLAKVLDVYEARLKEFKYLAGETFTLTDLHHIPVVQYL

LGTPTKKLFTERPRVNEWVAEVTKRPASQKILK

>Brapa10G001092.1

MVVKLYGQVTAACPQRVLLCLLEKEIEFEIIHIDLDTLEQKRPEHLLRQPFGQVPAIEDG

DFKLFESRAIARYYATKYAEQGTNLLGKSLEHRAIVDQWADVEVGYFNVLVHPLVMNLVI

KPRLGEECDAVLVEELKVKLGVVLDIYENQLASNRFLAGDEFTMADLTHMPAMGYLMRTN

INRIVKDRVNMNRWWEEVTARPAWKKLMKMAGF
